# Mechanical cues rewire lipid metabolism and support chemoresistance in epithelial ovarian cancer cell lines OVCAR3 and SKOV3

**DOI:** 10.1186/s12964-025-02144-9

**Published:** 2025-04-22

**Authors:** Martina Karasová, Maximilian Jobst, Denise Framke, Janice Bergen, Samuel Meier-Menches, Bernhard Keppler, Gunda Koellensperger, Jürgen Zanghellini, Christopher Gerner, Giorgia Del Favero

**Affiliations:** 1 Department of Food Chemistry and Toxicology, Faculty of Chemistry, University of Vienna, Währinger Str. 38-40, Vienna, 1090 Austria; 2 Doctoral School of Chemistry (DoSChem), Faculty of Chemistry, University of Vienna, Währinger Str. 42, Vienna, 1090 Austria; 3 Department of Analytical Chemistry, Faculty of Chemistry, University of Vienna, Währinger Str. 38, Vienna, 1090 Austria; 4 Department of Inorganic Chemistry, Faculty of Chemistry, University of Vienna, Währinger Str. 42, Vienna, 1090 Austria

**Keywords:** Ovarian cancer, Mechanotransduction, Mechanosensitive transcription factors, SREBP2, YAP1, Lipid metabolism, BOLD-100

## Abstract

Epithelial ovarian cancer (EOC) is one of the deadliest cancers in women, and acquired chemoresistance is a major contributor of aggressive phenotypes. Overcoming treatment failure and disease recurrence is therefore an ambitious goal. Ovarian cancer develops in a biophysically challenging environment where the cells are constantly exposed to mechanical deformation originating in the abdomen and shear stress caused by the accumulation of ascitic fluid in the peritoneal cavity. Therefore, mechanical stimulation can be seen as an inseparable part of the tumor microenvironment. The role of biomechanics in shaping tumor metabolism is emerging and promises to be a real game changer in the field of cancer biology. Focusing on two different epithelial ovarian cancer cell lines (SKOV3 and OVCAR3), we explored the impact of shear stress on cellular behavior driven by mechanosensitive transcription factors (TFs). Here, we report data linking physical triggers to the alteration of lipid metabolism, ultimately supporting increased chemoresistance. Mechanistically, shear stress induced adaptation of cell membrane and actin cytoskeleton which were accompanied by the regulation of nuclear translocation of SREBP2 and YAP1. This was associated with increased cholesterol uptake/biosynthesis and decreased sensitivity to the ruthenium-based anticancer drug BOLD-100. Overall, the present study contributes to shedding light on the molecular pathways connecting mechanical cues, tumor metabolism and drug responsiveness.

**Supplementary Information:**

The online version contains supplementary material available at 10.1186/s12964-025-02144-9.

## Introduction

During tumor progression, cancer cells are exposed to a constantly changing tumor microenvironment (TME). The TME comprises a complex interplay of different cell types and the extracellular matrix (ECM), which defines also its biomechanical properties [1]. To complete the process from tumor initiation, through tumor establishment to local and/or distant dissemination, cells must adapt their phenotype to different features of the TME [2, 3]. In addition to changes in biochemical composition, cells face a plethora of physical cues that drive cellular behavior, such as growth-induced solid stress within the tumor itself, increased stiffness of the surrounding matrix, and increased fluid pressure with possible concomitant fluid shear stress [2, 4].

Epithelial ovarian cancer (EOC) is a heterogeneous disease with at least 5 different major histological subtypes [5]. SKOV3 and OVCAR3 cell lines, commonly used as in vitro models of EOC, are classified as clear cell OC and high-grade serous OC, respectively [6]. It has been reported that ovarian cancer cells exhibit reduced cellular stiffness compared to their healthy counterparts [7], suggesting different cellular biomechanical properties between tumor and non-tumor cells. Ovarian cancer develops in a unique biophysical environment where fluid shear stress (SS) is of paramount importance [8]. SS induces spheroid formation and alters cytoskeletal organization in tumorigenic cells. Cells experiencing shear stress have been reported to form stress fibers, potentially facilitating peritoneal dissemination. Together with increased anchorage-independent survival, shear stress plays an important role in promoting invasion and metastasis [9, 10]. Importantly, fluid shear stress promotes the acquisition of cancer stem cell (CSC) characteristics and chemoresistance in ovarian cancer cells: this process involves the downregulation of microRNA-199a-3p, activation of epithelial-to-mesenchymal transition markers, the PI3K/Akt pathway, and multidrug transporters [11]. These findings highlight a critical role for physical cues in ovarian cancer progression and emphasize the need of research approaches including this dimension.

Understanding the reciprocal crosstalk between cell biomechanical properties (per se or altered by external TME) and cellular metabolism has been shown to be crucial for elucidating the mechanisms behind metabolic rewiring in cancer cells [12]. This bidirectional relationship includes the involvement of mechanosensitive transcription factor YAP1 (Yes-Associated Protein 1). Mechanical cues are capable of triggering its nuclear translocation and activation [13–15], which can additionally lead to the alteration of cellular metabolism in a YAP1-dependent manner [16, 17]. At the same time, metabolic rewiring itself may affect the mechanosensory apparatus of the cells. For example, lipid metabolism, especially cholesterol content in the plasma membrane, triggers changes in the activity of mechanosensitive ion channels [18] and the overall mechanosensory capacity of the plasma membrane [19]. Further, alteration of membrane structure or cytoskeletal dynamics and organization, can lead to possible modulation of YAP1 translocation potential [12, 20, 21].

Another metabolism-related regulator of YAP1 is the mevalonate pathway. It has been shown that mevalonate pathway promotes YAP/TAZ nuclear localization and activity. On the contrary, its inhibition by statins causes a marked accumulation of YAP/TAZ in the cytoplasm [22]. Upregulation of the mevalonate pathway has been associated with advanced-stage epithelial ovarian cancer, and the pharmacological inhibition of this metabolic route has shown promising results in reducing tumor growth [23]. This is also supported by evidence that mevalonate pathway inhibitors could be applied as potential therapeutic agents for ovarian cancer [24–26]. In addition to ovarian cancer, deregulation of the mevalonate pathway was already described in various cancers, including prostate, breast, lung, and hepatocellular carcinomas [27], and its main regulator is the Sterol Regulatory Element-Binding Protein 2 (SREBP2). The transcription factor SREBP2 promotes cancer stem cell-like properties, metastasis, and tumor growth by directly activating c-Myc in prostate cancer [28]. In colon cancer, the downregulation of SREBP2 inhibits tumor growth and initiation by altering cellular metabolism, reducing mitochondrial respiration, glycolysis, and fatty acid oxidation [29]. Additionally, SREBP2 has been found to contribute to cisplatin resistance by upregulating genes involved in cholesterol metabolism, such as LDLR, FDFT1, and HMGCR [30].

One of the most negative consequences of tumor plasticity is the capacity of cancer cells to acquire chemoresistance. Especially in ovarian cancer loss of therapeutic response remains a significant challenge, limiting treatment efficacy and patient survival. Several mechanisms of acquired chemoresistance have been proposed, including alterations in cellular metabolism [31–33], hence this raises the question whether physical stimuli developing at the tumor site could contribute to this phenomenon. Based on the fact, that key metabolic pathways are under the control of mechanosensitive transcription factors, such as YAP1 [13–15], HIF1α [34, 35] and SREBPs [36, 37], it is possible to envision a bidirectional relationship between the biomechanical properties of the TME and the metabolism of cancer cells [38]. With special focus on lipid homeostasis, this study is designed to contribute bridging the knowledge gap between response capacity of ovarian cancer cells to mechanical stimuli, metabolic adaptation and chemoresistance.

## Materials and methods

### Cell culture

The two commercially available human ovarian epithelial adenocarcinoma cell lines SKOV3 and OVCAR3, were obtained from the American Type Culture Collection (ATCC, Manassas, Virginia, USA). SKOV3 cells were cultured in McCoy’s 5a Medium Modified (Gibco, Thermo Fisher Scientific, Austria) supplemented with 10% fetal bovine serum (FBS; Gibco, Thermo Fisher Scientific, Austria) and 1% penicillin-streptomycin (P/S; Sigma-Aldrich, Austria), and OVCAR3 cells were cultured in RPMI-1640 Medium (Gibco, Thermo Fisher Scientific, Austria) supplemented with 20% FBS, 1% P/S and 1% insulin-transferrin-selenium (ITS; 100X, Gibco, Thermo Fisher Scientific), and maintained at 37 °C and 5% CO_2_. For Ca^2+^ free experiments, cells were maintained in Normal External Solution prepared without the divalent ion (NES; NaCl 140 mM, KCl 2.8 mM, MgCL_2_ 5 mM, Hepes 10 mM, glucose 10 mM) [39] supplemented with 10% and 20% FBS for SKOV3 and OVCAR3, respectively.

### Shear stress induction

Cells were seeded at a density of 10 × 10^3^/cm^2^ for SKOV3 and 50 × 10^3^/cm^2^ for OVCAR3 in 24, 12 or 6-well plates. Shear stress induction was carried out with the orbital shaker method [40] using MK3 control orbital shaker (IKA, Staufen, Germany). According to vessel type, a speed of 300 or 250 rpm was applied to achieve a comparable shear force (approx. 2 dynes/cm^2^), as previously described [41]. These values are in the range of physiological relevance for the peritoneal cavity (0–10 dynes/cm^2^ [9]), and align with literature describing the responsivity of ovarian cancer cells to mechanical cues (0.5-3 dynes/cm^2^ [9, 10, 41, 42]). The incubation times were set as short term (1–3 h) and long term (24 h), as already optimized for the two cell lines [43].

### Chemicals

PIEZO1 modulators, namely inhibitor GsMTx4 (cat. n. ab141871, Abcam, Cambridge, UK), and activator YODA1 (cat. n. SML1558, Sigma-Aldrich, Burlington, Massachusetts, United States) were dissolved in water or DMSO, respectively, as described by supplier [44–46]. Chemicals were used according to experimental design in concentrations 1 µM and 5 µM for YODA1 and 1 µM for GsMTx4.

The chemicals MβCD-Cholesterol (hereafter referred as Chol) and lovastatin (LOVA) were purchased from Sigma Aldrich (St. Louis, MI, USA), Enzo Farmingdale (New York, USA) respectively. Working concentrations Chol (10, 20 µg/ml), LOVA (0.1, 1, 10 µM) were chosen based on our data previously published [21]. The stock solutions of LOVA were dissolved in DMSO and MβCD-CHOL in ddH_2_O. DMSO, ddH_2_O, respectively, was used as solvent control, hereafter referred as ctrl.

BOLD-100 compound was synthesized as previously described [47]. The stock solutions of BOLD-100 were dissolved in DMSO and cells were treated with various concentrations (5-500 µM) for 48 h and DMSO was used as solvent control.

Cis-platin (cis-Pt) was dissolved in ddH_2_O to obtain a 5 mM stock solution. Final concentrations 10 µM and 20 µM for 48 h were used.

### Atomic force microscopy (AFM)

Evaluation of cellular stiffness was performed as previously described [48]. An AFM (JPK NanoWizard^®^ 4 XP, Bruker, Germany) coupled with an inverted Olympus IX73 optical microscope was used to measure Young’s modulus of living ovarian cancer cells. The setup enables for the direct overlay of optical imaging (phase contrast) and AFM mapping, allowing selective measurements of the cytoplasmic compartment. The cells were seeded into 34 mm tissue culture dishes (TPP Techno Plastic Products AG, Switzerland) and 48 h post-seeding shear stress experiments were conducted.

Cells were washed once with LCIS (37 °C) and imaged in LCIS. PFQNM-LC-A-CAL AFM tips (Bruker) with calibrated spring constant k ranging from 0.070 N/m to 0.090 N/m were used in QI™ mode. QI™ settings used are the following: Z-length: 1000 nm; applied force 0.2 nN; speed 100 μm/s [49]. Cells were selected using the phase contrast image and force maps were recorded in 25 μm x 25 μm areas. Cells were imaged for a maximum of 60 min to ensure stable cell viability. The cytoplasmic area was determined using the direct overlay of the optical image and the force map, force curves inside this area were selected and processed to obtain data of the Young’s modulus. The raw data was processed using JPK NanoWizard^®^ Data processing software. To set up the processing pipeline a representative force curve of the cell was used (Vertical deflection [nN] vs. Height [µm]). To start, the calculation of the average value of a section in the baseline and subtraction of this value from the whole curve, allowed for the baseline offset in vertical deflection to be removed. Vertical deflection [pN] was plotted against the vertical tip position [µm], by subtracting the cantilever deflection from the piezo height, to correct for the bending of the cantilever. For the determination of the reference force height a height value at 50% of the applied setpoint force was used. The elasticity fit model was used on the indentation data to calculate the Young’s modulus using the Hertz/Sneddon fit [50, 51] for a paraboloid tip shape with a 70 nm tip radius and a Poisson’s ratio value of 0.50 [52, 53]. This processing was then applied to all force curves in the selected cytoplasmic areas of the SKOV3 cells, and force curves were batch-analyzed in the selected cytoplasmic regions and median values of the Young’s modulus were determined [54].

### Live cell imaging – plasma membrane

The cellular plasma membrane was stained with CellMask™ Deep Red Plasma membrane stain (ThermoFisher), as previously described [55]. Briefly, cells were washed with pre-warmed DPBS and dye was applied in dilution 1:2000 and cellular nuclei were counterstained by Hoechst 33258 (ThermoFisher) in 1:1000 dilution for 30 min in 37 °C and 5% CO_2_. Thereafter, cells were washed and maintained in pre-warmed Live Cell Imaging Solution (LCIS, Molecular Probes, Life Technologies, Thermo Fisher Scientific, Waltham, USA) until microscopic analysis. Images were acquired with LSM Zeiss 710 equipped with ELYRA PS.1 system with a Plan Apochromat 63X/1.4 oil objective (zoom 1.5). Total membrane intensity analysis was performed with ZEN 2012 SP3 as mean intensity pro cell. For plasma membrane thickness, Z-stack option was used (1.67 μm interval). Image analysis was performed using ortho projection and thickness was measured at 3 different parts per cell. Membrane roughness was evaluated using the profile tool (signal intensity as function of distance). Cross-sections were made through whole cell and first 10 μm from cell borders were taken into final heatmap visualization.

### Membrane cholesterol quantification

Membrane cholesterol level was assessed as previously described [21]. Briefly, at the end of experiment, cells were fixed with 1% paraformaldehyde for 15 min in dark. After a washing step with PBS-A, filipin staining solution (SAE0088, Sigma Aldrich, St. Louis, MI, USA) at concentration 50 µg/ml was added for 1 h and incubated in the dark. Afterwards, cells were embedded in mounting medium without DAPI (F6182, Sigma Aldrich, St. Louis, MI, USA) and image acquisition was performed using Lionheart FX automated microscope (BioTek Instruments Inc., Winooski, VT, USA) with 10X magnification. At least 5 optical fields per well were taken and the mean signal intensity of each image was automatically calculated by Gen5 software (Gen5 ver. 3.08, BioTek Instruments Inc., Winooski, VT, USA) after background correction. All experiments were performed in at least 3 independent biological replicates.

### Membrane fluidity

Membrane fluidity assay was performed, as previously described [20, 21]. Briefly, cells were seeded in a black 96-well plate with clear bottom and 48 h post-seeding were loaded for 1 h with 1-pyrenedecanoic acid (10 µM, PDA, Thermo Fisher Scientific, Waltham, MA, USA). After excitation (344 nm) with a Cytation3 Imaging Multi-Mode Reader (BioTek, Winooski, VT, USA) fluorescence emission signals for PDA monomers (375 nm; *Im*) and excimers (470 nm; *Ie*) were measured. Membrane fluidity was expressed as ratio *Ie*/*Im*. Experiments were performed in at least 3 independent biological replicates and in 5 technical replicates.

### Immunofluorescence and image analysis

Subcellular localization of target proteins was assessed using an immunofluorescence approach. After shear stress and/or treatment incubation, cells were fixed with 3.7% paraformaldehyde for 10 min at RT and in the dark. For membrane proteins (PIEZO1 and LDLR), samples were blocked with 2% donkey serum (Sigma Aldrich) for 1 h at RT followed by application of primary antibodies (4 °C overnight). The permeabilization of plasma membrane by 0.2% TritonX-100 in PBS-A for 10 min at RT was applied afterwards. For cytoplasmic proteins (YAP1, Cav1, SREBP2, LATS1, p-LATS1) cells were permeabilized first, followed by blocking step for 1 h at RT. Primary antibodies were applied in dilutions ranging from 1:350 to 1:1000 (Table 1) and kept in 4 °C overnight. On the following day, the unbound antibodies were washed in 3 washing steps (0.05% TritonX-100 in PBS-A) and secondary antibodies against mouse/rabbit/goat were applied in dilution 1:1000. The actin cytoskeleton was counterstained by phalloidin green (cat. n. O7466, ThermoFisher) for Lionheart imaging or red (cat. n. A22283, ThermoFisher) for LSM imaging (according to the staining design) for 3 h at RT in the dark. Afterwards, further washing steps were performed and post-fixation with 3.7% paraformaldehyde was carried out. To quench remaining paraformaldehyde, solution of glycine/PBS was added for 5 min and finally, samples were washed with PBS-A for 5 min. Lastly, mounting medium with DAPI (cat. n. 104139, Abcam) was applied to stain cell nuclei.

**Table 1:** List of antibodies

Target	Cat. *N*.	dilution	Secondary antibody	Cat. *N*.
PIEZO1	ThermoFisher PA5_106296	1:400	Anti-Rabbit AF647	ThermoFisher A31573
LDLR	Santa Cruz sc-18,823	1:350	Anti-Mouse AF647	ThermoFisher A31571
YAP1	Abcam ab52771	1:1000	Anti-Rabbit AF647, Anti-Rabbit AF488	ThermoFisher A31573 ThermoFIsher A21206
Caveolin-1	Abcam ab211503	1:750	Anti-Goat, AF647	ThermoFisher A21447
SREBP2	Biotechne AF7119	1:500	Anti-Goat, AF647	ThermoFisher A21447
LATS1	Proteintech 17049-1-AP	1:500	Anti-Rabbit AF647	ThermoFisher A31573
Phospho-LATS1 (Thr1079)	Proteintech 28998-1-AP	1:400	Anti-Rabbit AF647	ThermoFisher A31573

For image acquisition of actin, YAP1, PIEZO1, LDLR, LATS1 and p-LATS1 the Lionheart FX automated microscope (BioTek Instruments Inc., Winooski, VT, USA) was used with 20X magnification. For every sample, at least 4 optical fields were randomly chosen resulting in minimal *n* = 16 optical fields from at least 3 independent biological replicates. For SREBP2, YAP1 and Caveolin-1, at least 5 images per sample were acquired with LSM Zeiss 710 equipped with ELYRA PS.1 system with a Plan Apochromat 63X/1.4 oil objective (zoom 1.5) and at least 50 cells were analyzed.

Image analysis was performed using Gen5 software (Gen5 ver. 3.08, BioTek Instruments Inc., Winooski, VT, USA) for images acquired with Lionheart FX automated microscope. The mean signal intensity of the target protein was automatically calculated in both cytoplasmic and nuclear regions. Total protein level was calculated as sum of values obtained in cytoplasmic and nuclear region. Nuclear translocation of transcription factors was expressed as nuclear to cytoplasmic ratio (n/c ratio).

For actin cytoskeleton analysis, phalloidin green staining was used. The total signal intensity and signal area was assessed using Gen5 software (Gen5 ver. 3.08, BioTek Instruments Inc., Winooski, VT, USA).

For LSM Zeiss 710 acquired images, manual analysis in ZEN (black edition) 2.3 system was conducted. For SREBP2 and YAP1, single cell analysis was performed as mean intensity of both cytoplasmic and nuclear ROIs (region of interest). The total protein level and n/c ratio were calculated as described above. At least 4 cells were analyzed per optical field, which makes at least 60 cells analyzed per condition. For the analysis of nuclear morphology, cell nuclei were stained with DAPI and imaged by LSM Zeiss 710 equipped with ELYRA PS.1 system with a Plan Apochromat 63X/1.4 oil objective (zoom 1.5). Quantification was performed using the imageJ 1.53a software and the parameters circularity (4 *π * (area/perimeter^2^)), aspect ratio (major axis/minor axis), and solidity (area/convex area) were determined [56]. In total *n* ≥ 60 cells per condition taken from at least 3 independent cell preparations (biological replicates) were evaluated.

### Intracellular calcium quantification

For intracellular calcium kinetics measurement, cells were seeded in µ-Plate 96 Well Black ibiTreat (Ibidi GmbH, Gräfelfing, Germany), as previously described [44]. To assess the basal responsiveness of two OC cell lines to modulation of PIEZO1 activity, both SKOV3 and OVCAR3 were treated with YODA1. For long term PIEZO1 activation, 1 µM YODA1 for 24 h was used, for acute PIEZO1 activation 5 µM for 25 min was applied. In order to check additive effect, both pre-treatment and acute treatment were combined. After removal of the incubation media 75 µL of Fluo-4 solution were added (1:1000 Fluo-4 AM, 1:100 PowerLoad) in Live Cell Imaging Solution (LCIS, cat. n. F14217, Invitrogen). After washing, the [Ca^2+^]_i_ levels were measured using a multi-detector microplate reader Synergy H1 Hybrid (BioTek, Bad Friedrichshall, Germany) at different timepoints [0-1-2 min] as fluorescence signal [484, 525 nm], to establish a base level. Afterwards cells were quickly incubated with YODA1 (5 µM) and measured again in 1 min intervals. The kinetic calcium signal was quantified following these equations: for the initial increase after one minute: increase [%] = 100 * ([Ca^2+^ t1] – [Ca^2+^ basal]) / [Ca^2+^ basal] and for the total increase after 23 min: increase [%] = 100 * ([Ca^2+^ t23] – [Ca^2+^ basal]) / [Ca^2+^ basal]. Per condition at least *n* ≥ 12 technical replicates were performed from 3 different cell preparations (biological replicates).

### Metabolomic assay

Cells were seeded into 6-well plates in three biological replicates at numbers 7.5 × 10^3^/cm^2^ for SKOV3 and 37.5 × 10^3^/cm^2^ for OVCAR3 and cultured for 48 h. Then, SKOV3 cells were treated with Chol (10 µg/ml) and OVCAR3 with LOVA (1 µM) and shear stress (250 rpm, 24 h) was applied simultaneously. Cells were quickly washed 1x with PBS and 80 µL of extraction solvent (ethanol/phosphate buffer 85:15 v: v) were added to each well. Cells were frozen in liquid nitrogen and 3 freeze/thaw cycles were performed in total. Extracts were transferred into Eppendorf tubes and stored at − 80 °C. An aliquot of 40 µL of each extract (2 × 20 µL) was used for loading. Targeted metabolomics experiments of cellular extracts were conducted by the MxP^®^ Quant 500 Kit (Biocrates Life Sciences AG, Innsbruck, Austria). Measurements were carried out using LC-MS/MS and flow injection (FIA)-MS/MS analyses on a Sciex 6500 + series mass spectrometer coupled to an ExionLC AD chromatography system (AB Sciex, Framingham, MA, USA), utilizing the Analyst 1.7.1 software with hotfix 1 (also AB SCIEX). All required standards, quality controls and eluents were included in the kit, as well as the chromatographic column for the LC-MS/MS analysis part. Phenyl isothiocyanate (Sigma-Aldrich, St. Louis, USA) was employed for derivatization of amino acids and biogenic amines according to the kit manual. Preparation of the measurement worklist as well as data validation and evaluation were performed with the software supplied with the kit (MetIDQ-Oxygen-DB110-3005, Biocrates Life Sciences). A total of 237 metabolites showed signal intensities within the quantification window and were further evaluated.

### WST-1 assay

Following treatments with MβCD-Cholesterol (10, 20 µg/ml), lovastatin (0.1, 1, 10 µM) for 24 h, a WST1 assay was performed as previously described [20, 21]. Briefly, cells were seeded in 96-well plate and treated as desired. At the endpoint, cells were washed with pre-warmed PBS 100 µL and WST-1 solution (1:20 in medium) was added to each well and incubated for 30 min at 37 °C, 5% CO_2_. The absorbance was measured using a multi-detector microplate reader Synergy H1 Hybrid (BioTek, Bad Friedrichshall, Germany) with the wavelength 440 nm. The data are shown as percentage of respective solvent control. The experiments were performed at 3 technical replicates and 3 biological replicates.

### Crystal Violet assay

Cells were seeded in triplicate in 96-well plate (10 × 10^3^/well and 50 × 10^3^/well for SKOV3 and OVCAR3, respectively) and following BOLD-100 treatment (concentrations ranging from 10 to 500 µM) for 48 h, cell viability, expressed as cellular biomass, was assessed performing a crystal violet assay. As previously described [57, 58], cells were washed with pre-warmed PBS and fixed with cold ethanol (99%) for 10 min. Afterwards, staining solution of 0.1% crystal violet was applied for 10 min. Excess staining solution was aspired and washed several times with H_2_O and cells were lysed with de-staining solution (1% acetic acid in 99% ethanol) for 10 min.

For shear stress experiments, cells were seeded in 12-well plates (for SKOV3 7,5 × 10^3^/cm^2^, for OVCAR3 35 × 10^3^/cm^2^) and shaken for timepoint indicated by each experiment. The treatment with LOVA and GsMTx4 was applied simultaneously with shear stress. BOLD-100 treatment was added afterwards for another 48 h. For co-treatment of BOLD-100 and LOVA experiments in static conditions, cells were seeded in 96-well plate (for SKOV3 10 × 10^3^/well, for OVCAR3 40 × 10^3^/well) and co-treatment (BOLD-100 in concentrations 5, 10, 50, 100, 200 µM and LOVA in concentration 1 µM) was applied for 48 h.

At the end of incubation times, cells were fixed and stained with crystal violet, as described above. Absorbance (595 nm) was measured using plate reader Synergy H1 Hybrid (BioTek, Bad Friedrichshall, Germany). At least three independent cell replications and three technical replications were measured. The results are expressed as percentage of solvent control.

### Caspase3/7 assay

Following treatment with BOLD-100 in SKOV3 and OVCAR3 cell lines, cells were seeded in 96-well plate (10 × 10^3^/well, 50 × 10^3^/well, respectively). 24 h post seeding, cells were treated with BOLD-100 in concentration range 50–500 µM for 48 h, as induction of Caspase3/7 was shown to occur in higher concentrations [59]. Treatment with cis-Pt in concentrations 10 µM and 20 µM for 48 h was used as positive control for Caspase3/7 activation [60]. CellEvent™ Caspase-3/7 Green ReadyProbes™ Reagent (cat.n. R37111, Invitrogen, Oregon, USA) was used according to manufactures’ instruction. Briefly, 2 drops of ready-to-use solution and Hoechst 33342 solution (cat.n. 62249, Thermo Scientific, Waltham, MA, USA) in dilution 1:2000 was added into 1 ml of complete media. 50 µl/well of staining solution was added to cells for 30 min in 37 °C/ 5% CO_2_. Afterwards, staining solution was aspired, and cells were washed with pre-warmed PBS. For live cell imaging Lionheart FX automated microscope (BioTek Instruments Inc., Winooski, VT, USA) was used with 10X magnification. For every sample, 3 optical fields were randomly chosen resulting in minimal *n* = 9 optical fields from at least 3 independent biological replications. Image analysis was performed using Gen5 software (Gen5 ver. 3.08, BioTek Instruments Inc., Winooski, VT, USA). Results are shown as percentage of Caspase3/7 signal of untreated cells.

### Statistical analysis

All datasets were statistically evaluated and plotted using the software OriginPro 2023b version 10. Data variance was tested using f-test and for cut-off value *p* < 0.05, Student’s t-test for normal distribution was applied. For *p* < 0.05 indicating unequal variance, Welch correction was assumed. Multiparametric comparisons were assessed by one-way ANOVA test. Significant differences among treatment groups were identified using a cut-off of 0.05 (p-value).

## Results

### Mechanosensory apparatus in EOC: cell membrane

**Fig. 1:**
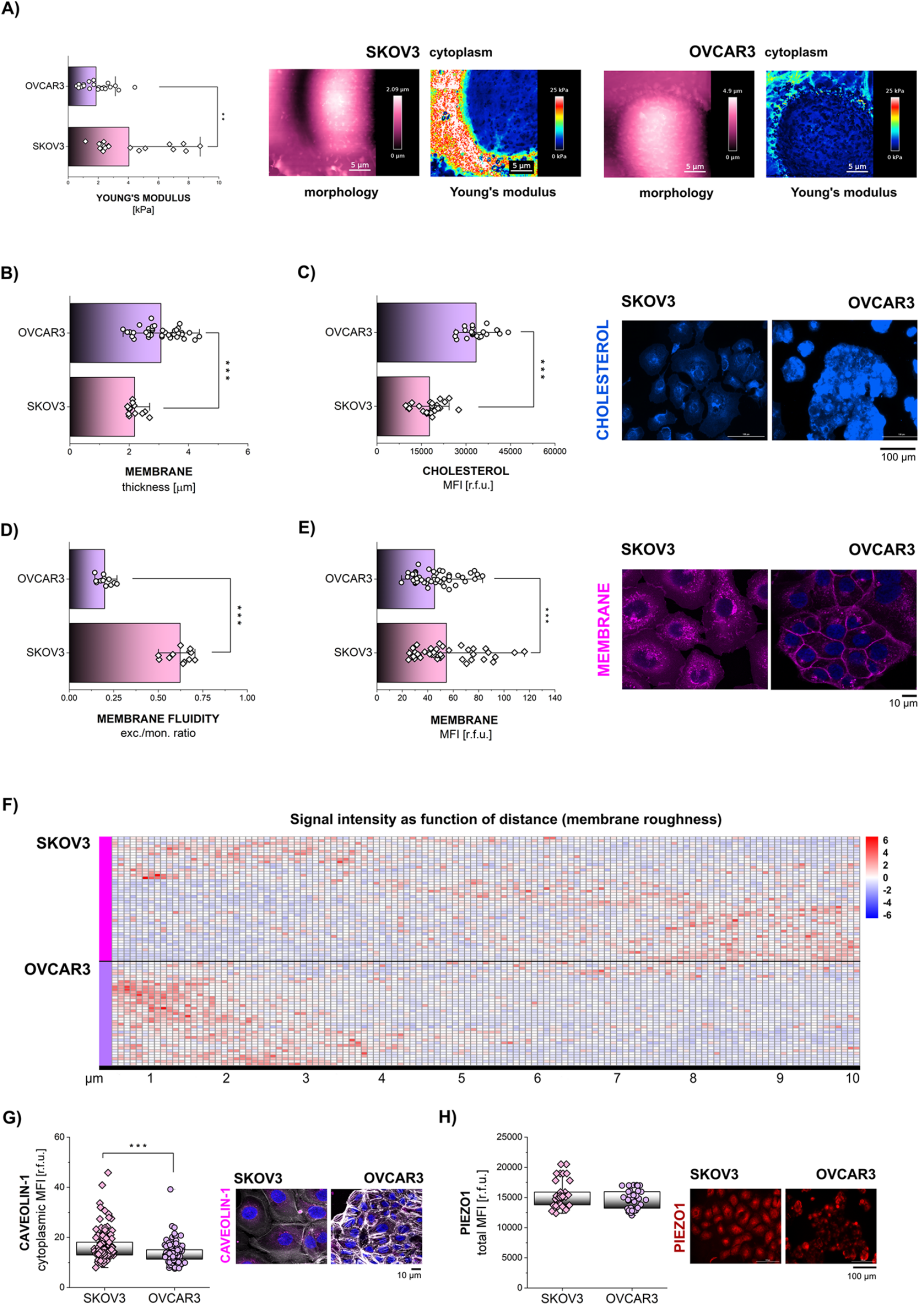
Mechanosensory apparatus in EOC: cell membrane **(A)** Quantification of the Young’s modulus [kPa] median per ROI for cytoplasmic areas of the SKOV3 and OVCAR3 cells with representative AFM maps. The height is shown in pink, the Young’s modulus depicting values between 0–25 kPa is shown in blue-to-red. **(B)** Plasma membrane thickness [µm] measured at 3 different points per cell. **(C)** Cholesterol quantification [r.f.u.] with representative images of plasma membrane cholesterol signal depicted in blue. **(D)** Membrane fluidity [PDA excimers/monomers ratio]. **(E)** Membrane intensity [r.f.u.] with representative images of PM appearance in magenta, nuclei in blue. **(F)** Membrane roughness expressed as signal intensity of the PM staining as function of distance. Segments of 10 μm were taken into final heatmap visualization. Rows depict single cells, *n* > 37 for each cell line. **(G)** Cytoplasmic expression of Caveolin-1 [r.f.u.] with representative images. Caveolin-1 is depicted in magenta, actin cytoskeleton in white and nuclei in blue. **(H)** Total protein level of PIEZO1 [r.f.u.] and representative images (depicted in red). All experiments were performed in at least 3 independent biological replicates. Statistical significance calculated with t-test and shown as * *p* < 0.05, ** *p* < 0.01, *** *p* < 0.001

To gain insights into the biomechanical sensory apparatus of SKOV3 and OVCAR3, a panel of assays was performed to profile the similarities and differences between the two cell lines. Cellular stiffness belongs to the physical hallmarks of cancer [61] and can give precious information on the mechanical plasticity of the cells. Using atomic force microscopy, Young’s modulus of SKOV3 and OVCAR3 cells was measured in the cytoplasmic compartment. Here, SKOV3 showed a higher stiffness than OVCAR3 (Fig. 1A). Further, as the plasma membrane (PM) is the first interface towards extracellular milieu, including mechanical cues, PM properties and composition were compared in the two cell lines. Aligning with lower Young’s modulus, morphometric characterization of the PM returned that OVCAR3 have a thicker membrane (Fig. 1B), which corresponds to a higher membrane cholesterol content (Fig. 1C). Cholesterol acts as a bidirectional regulator of membrane fluidity and, in our models, OVCAR3 cells with high cholesterol levels showed reduced plasma membrane fluidity in comparison to SKOV3 (Fig. 1D). As PM is not flat and possess the capacity to stretch in the response to physical cues [62–64], PM roughness, namely its topography resulting from invaginations and curvatures, might provide precious hints about the adaption potential of the compartment. Mapping signal membrane intensity toward distance, supported the identification of high intensity areas toward the whole sampling length in SKOV3, possibly mirroring higher PM folds or high membrane roughness (Fig. 1E-F). Looking for structural features potentially accounting for this phenotype, the major component of the mechanosensitive coated pits [65], caveolae-binding protein Caveolin-1 (CAV-1) was quantified. Coherently with the roughness of the membrane, SKOV3 displayed higher expression of CAV-1 compared to OVCAR3 cells (Fig. 1G). As mechanosensitive ion channels are expressed in some tissues in close proximity of CAV-1 [66], expression of PIEZO1 channels was also evaluated. For the mechano-gated ion channels, SKOV3 and OVCAR3 returned similar values of mean signal intensities (Fig. 1H).

### Mechanosensory apparatus in EOC: PIEZO1 activity

**Fig. 2:**
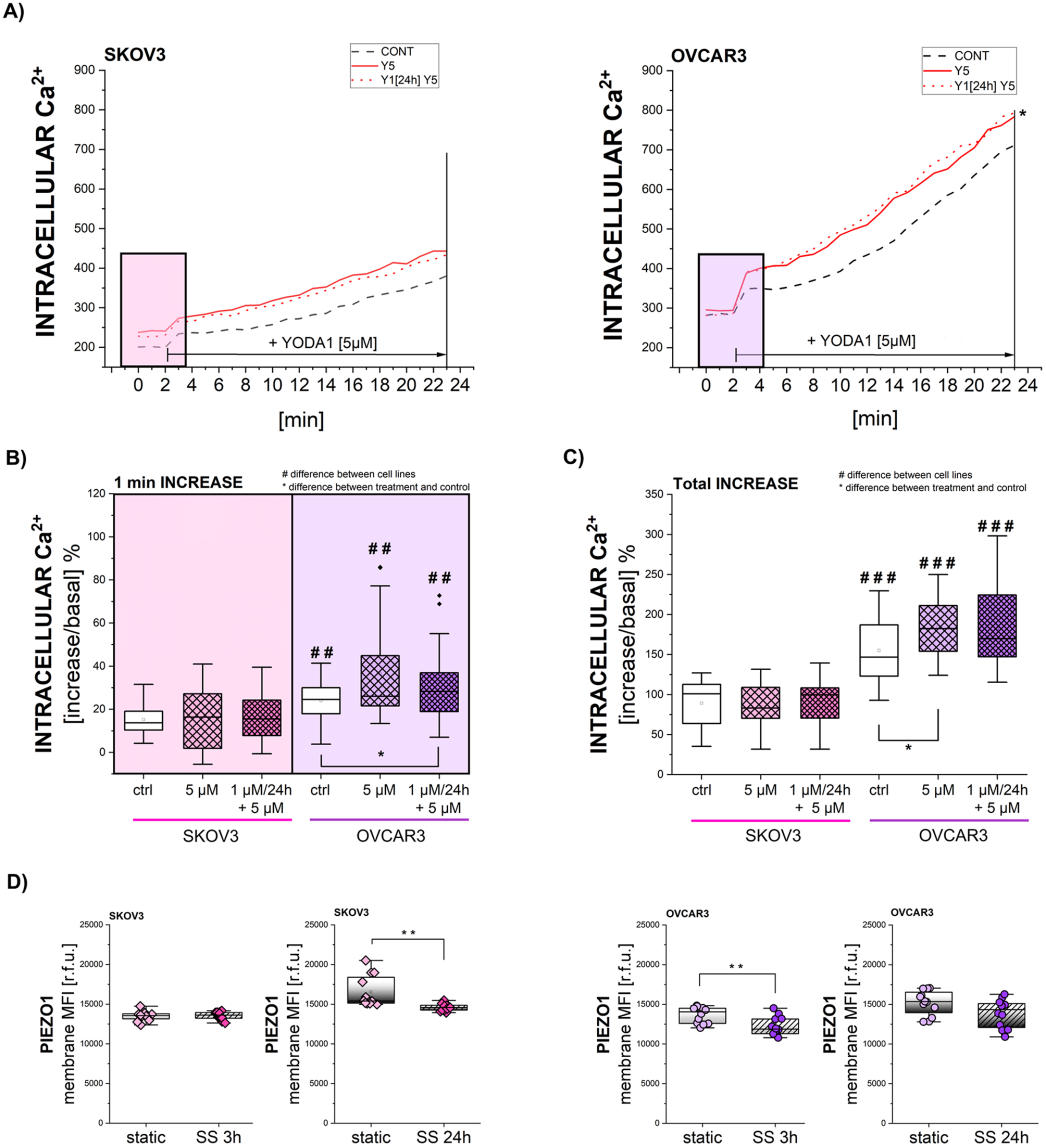
Mechanosensory apparatus in EOC: PIEZO1 activity **(A)** Illustrative curves of intracellular Ca^2+^ levels as function of time with the first increase after YODA1 (5 µM) treatment highlighted in box **(B)** Immediate increase (1 min) in intracellular Ca^2+^ levels in cells treated with YODA1 (5 µM) with or without pre-treatment with YODA1 (1 µM) for 24 h. Increase is expressed as percentage of increase/basal levels ratio. * represents the difference between treatment and respective control, # represents differences between cell lines. **(C)** Total increase (23 min) in intracellular Ca^2+^ levels in cells treated with YODA1 (5 µM) with or without pre-treatment with YODA1 (1 µM) for 24 h. Increase is expressed as percentage of increase/basal levels ratio. * represents the difference between treatment and respective control, # represents differences between cell lines. **(D)** Quantification of PIEZO1 expression [r.f.u.] at the plasma membrane in cells exposed to SS for 3 h and 24 h and in respective static controls. All experiments were performed in at least 3 independent biological replicates. Statistical significance calculated with t-test and one-way ANOVA, and shown as */# *p* < 0.05, **/## *p* < 0.01, ***/### *p* < 0.001

To start shedding light on the response capacity of SKOV3 and OVCAR3 to mechanical stimuli, experiments were performed to explore the function of PIEZO1 channels. Measurements of intracellular calcium levels were acquired in control conditions and after chemical activation of PIEZO1 by agonist molecule YODA1 (5 µM). Persistent activation, as for continuous SS, was simulated with 24 h chemical pre-conditioning with 1 µM YODA1 [45] and followed by acute activation with 5 µM YODA1. With these experimental layouts, the Ca^2+^ kinetic profiles of SKOV3 remained unaltered regardless of the treatment, whereas significant variations could be observed in OVCAR3 cells, expressed as Ca^2+^ increase/ Ca^2+^ basal levels ratio (Fig. 2A-C). OVCAR3 responded to acute PIEZO1 activation by increasing Ca^2+^ levels after 1 min, a response which consolidated in a significant increase in comparison to controls after 20 min of treatment (Fig. 2B-C). Response capacity of OVCAR3 to acute stimulation, was maintained even after 24 h pre-exposure to YODA1, with a significant increase in comparison to controls after 1 min re-exposure to the agonist (Fig. 2A-C). To get further insight into how PIEZO1 is modulated by mechanical stimuli, shear stress protocols were applied. After 3 h and 24 h shear stress stimulation, immunofluorescence analysis of PIEZO1 expression revealed significant differences among the response profile of the two cell types. In OVCAR3, PIEZO1 expression at the PM decreased after 3 h SS and this reduction was still visible, even if not significant, after 24 h SS. In contrast, in SKOV3, PIEZO1 levels remained unchanged after 3 h SS, but significantly decreased after long-term stimulation (Fig. 2D). In order to explore if the signal decrease at the PM level could be related to internalization, PIEZO1 signal was quantified also in the cytoplasmic compartment, however no changes could be observed at this level (Suppl. Figure S1).

### Shear stress stimulation in EOC: cell morphometric adjustment

Among the possible consequences of PIEZO1-mediated Ca^2+^ influx [67] there are actin stress fiber formation and cytoskeleton reorganization [68]. In agreement, it has been reported that the actin cytoskeleton is remodeled after SS treatment in various cell types, including ovarian cancer cells [9, 69–71]. Following up on the differences detected for PIEZO1 activation in OVCAR3 and SKOV3, experiments were performed to verify the functional response on the actin cytoskeleton after short-term (3 h) and long-term (24 h) mechanical stimulation.

**Fig. 3:**
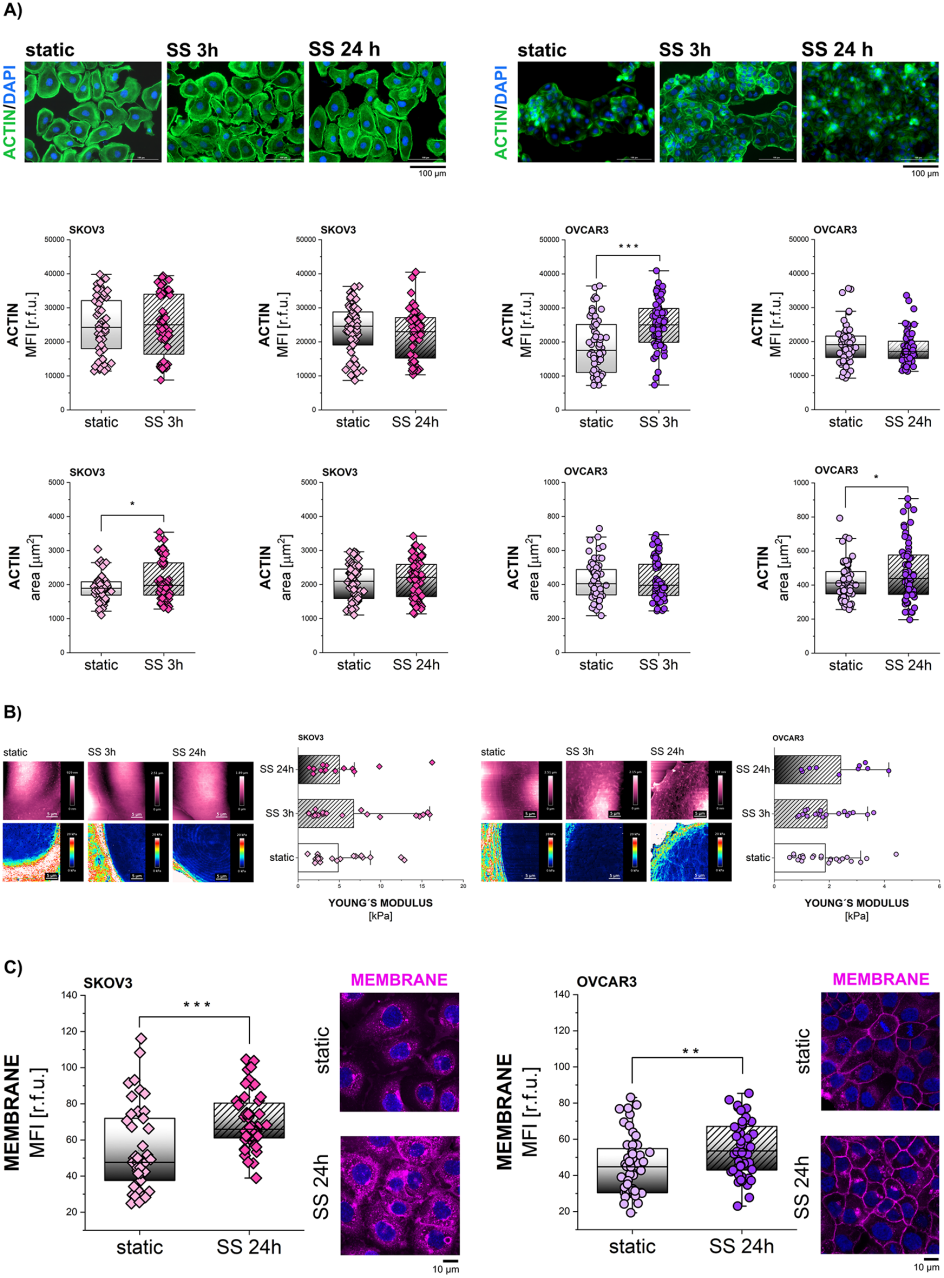
Shear stress stimulation in EOC: cell morphometric adjustment. **(A)** Quantification of actin cytoskeleton of cells exposed to SS (3 h and 24 h) and respective static controls. Actin intensity is expressed as MFI [r.f.u.], cytoplasmic area is expressed in [µm^2^]. In representative images, actin is depicted in green, nuclei in blue. **(B)** Quantification of the Young’s modulus median per ROI [kPa] for cytoplasmic areas of the SKOV3 and OVCAR3 cells exposed to SS (3 h and 24 h) and respective static controls. Representative AFM maps show the height depicted in pink, the Young’s modulus representing maps depict values between 0–20 kPa (shown in blue-to-red). **(C)** Quantification of PM intensity [r.f.u.] in cells exposed to 24 h SS and in respective static controls. In representative images, PM depicted in magenta, nuclei in blue. Statistical significance calculated with t-test and shown as * *p* < 0.05, ** *p* < 0.01, *** *p* < 0.001

Actin cytoskeleton rearranged significantly in SKOV3 within 3 h stimulation, with a significant increase of actin-covered area and no variation of the actin signal intensity (Fig. 3A). In contrast, OVCAR3 rapidly formed stress fibers after 3 h SS; this phenotype returned increased total cytoplasmic actin signal and increase in cell area after 24 h exposure to mechanical cues (Fig. 3A). In parallel, AFM experiments were performed to gain insights on cytoplasmic stiffness. Even if a trend in the adaptation of the Young’s modulus could be observed mirroring the area adjustment, this was not significant for SKOV3 and OVCAR3 after 3 h and 24 h mechanical stimulation in comparison to controls (Fig. 3B). To verify if the adaptation of the actin cytoskeleton could find parallel on the membrane adjustment, cell imaging experiments were performed focusing on the PM compartment. After 24 h stimulation, SS resulted in an increased PM signal in both cell lines (Fig. 3C). Taken together, SKOV3 and OVCAR3 display some differences (actin cytoskeleton rearrangement) and similarities (effect on PM) in response to mechanical stimulation induced by shear stress.

### Shear stress stimulation in EOC: mechanosensitive regulation of SREBP2

**Fig. 4:**
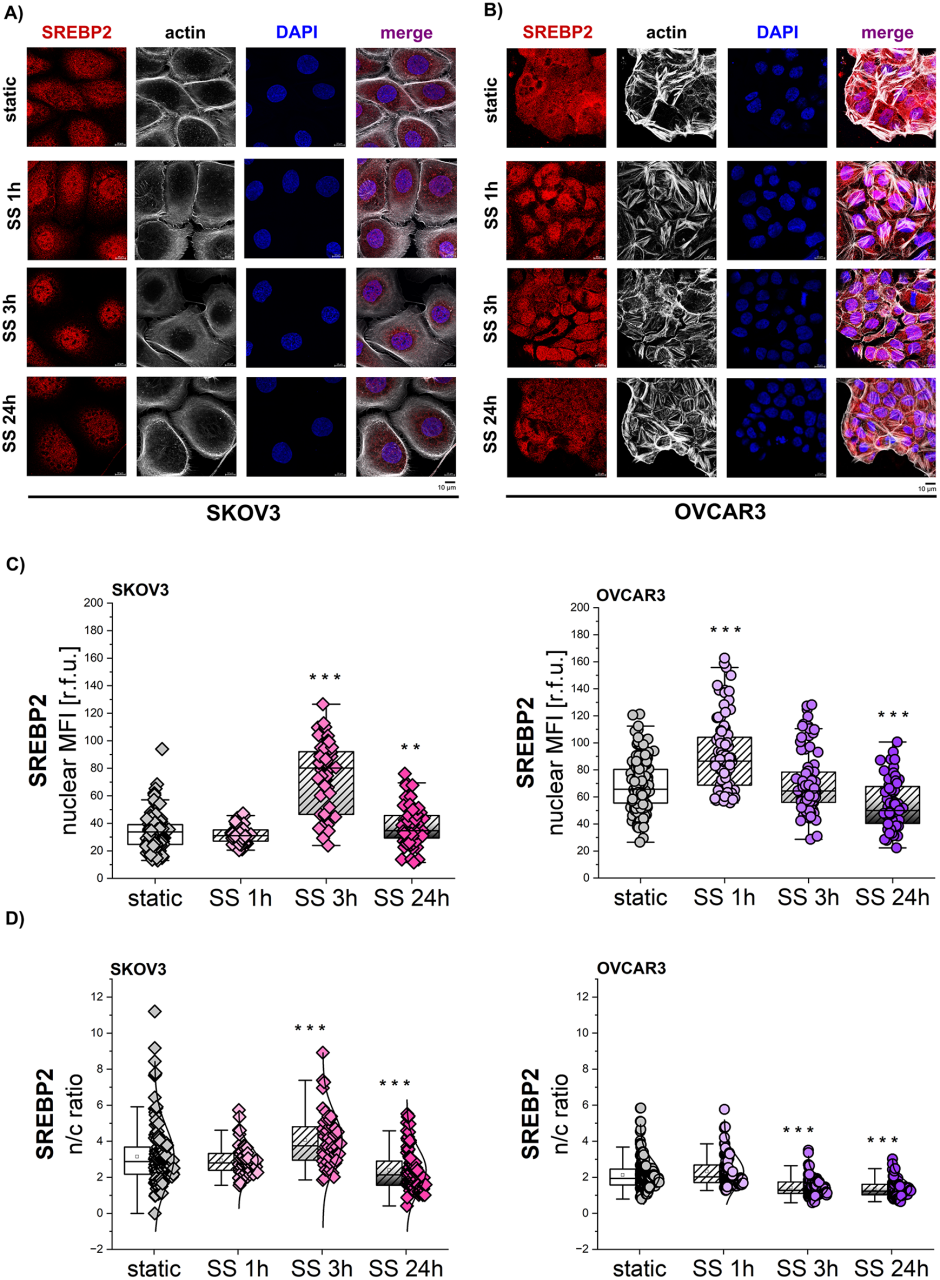
Shear stress stimulation in EOC: mechanosensitive regulation of SREBP2 **A** + **B**) Representative images of SREBP2 depicted in red, actin cytoskeleton in white and nuclei in blue. **C)** Nuclear protein levels of SREBP2 [r.f.u.] in cells exposed to 1 h, 3 h and 24 h of SS and in respective static controls. **D)** SREBP2 nuclear/cytoplasmic ratio (n/c ratio) in cells exposed to 1 h, 3 h and 24 h of SS and in respective static controls. All experiments were performed in at least 3 independent biological replicates. Statistical significance calculated with t-test and shown as * *p* < 0.05, ** *p* < 0.01, *** *p* < 0.001

As the mammalian plasma membrane is composed of 30–40% of cholesterol and SS induced increase in PM signal intensity (Fig. 3C), the focus was moved on the regulation of cholesterol biosynthesis upon mechanical stimulation. Cholesterol biosynthesis and uptake are under the transcriptional control of SREBP2. When cholesterol levels are restored, SREBP2 is excluded from the nucleus and degraded [72, 73]. Of the same family of transcription factors, SREBP1 was previously described to regulate lipid metabolism in response to the stiffness of the extracellular matrix [36]. Taking this as a starting point, an involvement of SREBP2 was postulated. It was shown that translocation of transcription factors upon mechanical stimuli can be as fast as in 30 min [74]. Therefore, SS was applied for 1 h, 3 h and 24 h to observe possible translocation kinetics of SREBP2 (Fig. 4A-B). Three parameters were quantified: nuclear localization of the protein, its nuclear-to-cytoplasmic ratio (n/c ratio) and total protein level/cell. After 1 h of shear stress no changes could be observed for SREBP2 in SKOV3 cells (Fig. 4C-D, suppl. Figure S2). A significant increase in nuclear (217% of static control) and total protein level (205% of static control), as well as in n/c ratios (125% of static control) was visible after 3 h of SS. After 24 h SS a more moderate increase was visible in the nuclear signal and the n/c ratios significantly decreased in comparison to controls (Fig. 4C-D, suppl. Figure S2).

In OVCAR3 significant increase (approximately 36%) could be measured for both nuclear and total levels of SREBP2 already after 1 h SS; data collected after 3 h incubation describe a return of the nuclear/total signal to control levels and a significant decrease of n/c signal ratio (Fig. 4C-D, suppl. Figure S2). After 24 h SS, nuclear, total cell signals and n/c ratios for the TF were significantly lower in comparison to controls (Fig. 4C-D, suppl. Figure S2). Taken together, data suggested different kinetics of SREBP2 re-localization in SKOV3 and OVCAR3 cell lines, with a slightly faster response for OVCAR3 in comparison to SKOV3 (Suppl. Figure S2B).

### Shear stress stimulation in EOC: impact on cholesterol biosynthesis and uptake

**Fig. 5:**
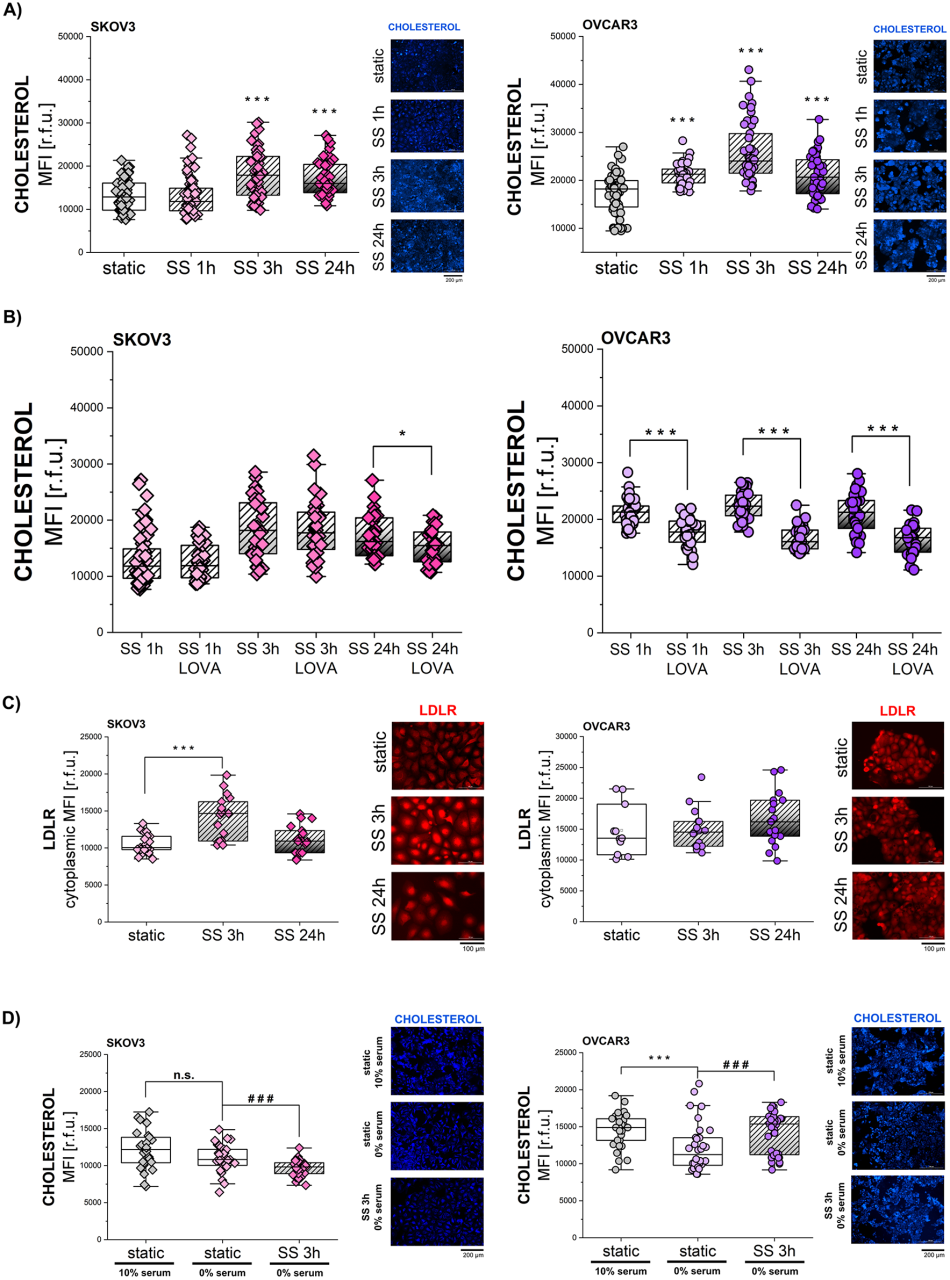
Shear stress stimulation in EOC: impact on cholesterol biosynthesis and uptake **(A)** Quantification of cholesterol level [r.f.u.] in cells exposed to 1 h, 3 h and 24 h of SS and in respective static control. Representative images show cholesterol at plasma membrane depicted in blue. **(B)** Quantification of cholesterol level [r.f.u.] in cells exposed to 1 h, 3 h and 24 h of SS in presence of LOVA (1 µM) for 1, 3–24 h, respectively. * depicts significant differences between LOVA treatment and solvent control **(C)** Quantification of LDLR expression [r.f.u.] at the plasma membrane in cells exposed to 3 h and 24 h of SS and in respective static controls. In illustrative images LDLR depicted in red. **(D)** Quantification of cholesterol level [r.f.u.] in cells exposed to 3 h SS and in respective static controls in presence of 10% serum (control) or 0% serum. * depicts significant differences between 10% serum (control) and 0% serum. # depicts difference between static and SS. Representative images show cholesterol at plasma membrane depicted in blue. All experiments were performed in at least 3 independent biological replicates. Statistical significance calculated with t-test and shown as */# *p* < 0.05, **/## *p* < 0.01, ***/### *p* < 0.001

Once showed that SS triggers the translocation of SREBP2 in SKOV3 and OVCAR3, further experiments were performed to verify if the movement of the TF also triggers a functional outcome, namely cholesterol synthesis via mevalonate pathway. Firstly, cholesterol level was measured after 1 h, 3 h and 24 h of SS. In SKOV3, an increase in membrane cholesterol levels could be observed starting after 3 h SS (increase about 38%) and this effect was maintained even after 24 h incubation. In OVCAR3, significant increase (about 21%) was observed already after 1 h SS and the maximal response (mean increase of 48%) was reached after 3 h SS (Fig. 5A).

To explore the hypothesis that SS application could lead to an induction of endogenous cholesterol biosynthesis, cells were co-treated with the cholesterol biosynthesis inhibitor lovastatin (LOVA). Absence of toxicity was confirmed in the concentration range 0.1–10 µM for LOVA (Suppl. Figure S3 A) and concentration of 1 µM was chosen for combination with shear stress (1 h, 3 h and 24 h). In SKOV3 cells, LOVA had no effect on cholesterol level at 1 h and 3 h time points and led to a slight decrease after 24 h co-incubation (Fig. 5B). In OVCAR3, LOVA decreased cholesterol level at all indicated time points compared to the solvent control (Fig. 5B).

Building on this, a connection between mechanical driven phenotype and the capacity of ovarian cells to take up lipids from the extracellular environment was postulated. This is of great pathophysiological relevance as SREBP2 initiates the transcription of genes involved in cholesterol biosynthesis and uptake. Moreover, ovarian cancer exhibits a distinct metastatic preference for the fat-rich omentum within the peritoneal cavity [75–78]. This unique biology of ovarian cancer metastasis, characterized by peritoneal dissemination and superficial invasion, poses treatment challenges and contributes to its low cure rate [78]. To examine the origin of elevated cholesterol levels in SKOV3, further experiments were conducted. Firstly, the behavior of the low-density lipoprotein receptor (LDLR) was investigated after 3 h and 24 h SS. SKOV3 cells showed a significant increase in membrane LDLR expression after 3 h SS (Fig. 5C). Prolonged exposure to physical cues (24 h SS) resulted in a return of LDLR levels comparable to static controls. In contrast, OVCAR3 cells did not respond to shear stress in terms of modulation of LDLR expression (Fig. 5C). These results suggested a different dependence on cholesterol uptake vs. biosynthesis in SKOV3 and OVCAR3 cells. To further follow this hypothesis, both cell lines were exposed to 3 h of shear stress in control (10% serum) and lipid-depleted (0% serum) conditions. After mechanical stimulation, membrane cholesterol levels were evaluated. SKOV3 cells did not increase cholesterol levels after 3 h of SS in 0% serum conditions as they did in standard cholesterol-containing culture medium (Fig. 5A and D). However, OVCAR3 cells increased their cholesterol levels independently of the extracellular cholesterol supply (Fig. 5D). These data supported the view that upon application of shear stress SKOV3 cells seem to preferentially take up cholesterol from the extracellular environment whereas OVCAR3 cells more efficiently boost cholesterol biosynthesis. To additionally explore the mechanistic link between shear stress application and cholesterol accumulation, experiments were performed with mechanosensitive channel agonist YODA1: no cholesterol accumulation was observed upon direct PIEZO1 chemical activation (Suppl. Figure S3B) indicating that the opening of the ion channel alone might not be sufficient for SS-induced cholesterol accumulation.

### Shear stress stimulation in EOC: mechanosensitive regulation of YAP1

**Fig. 6:**
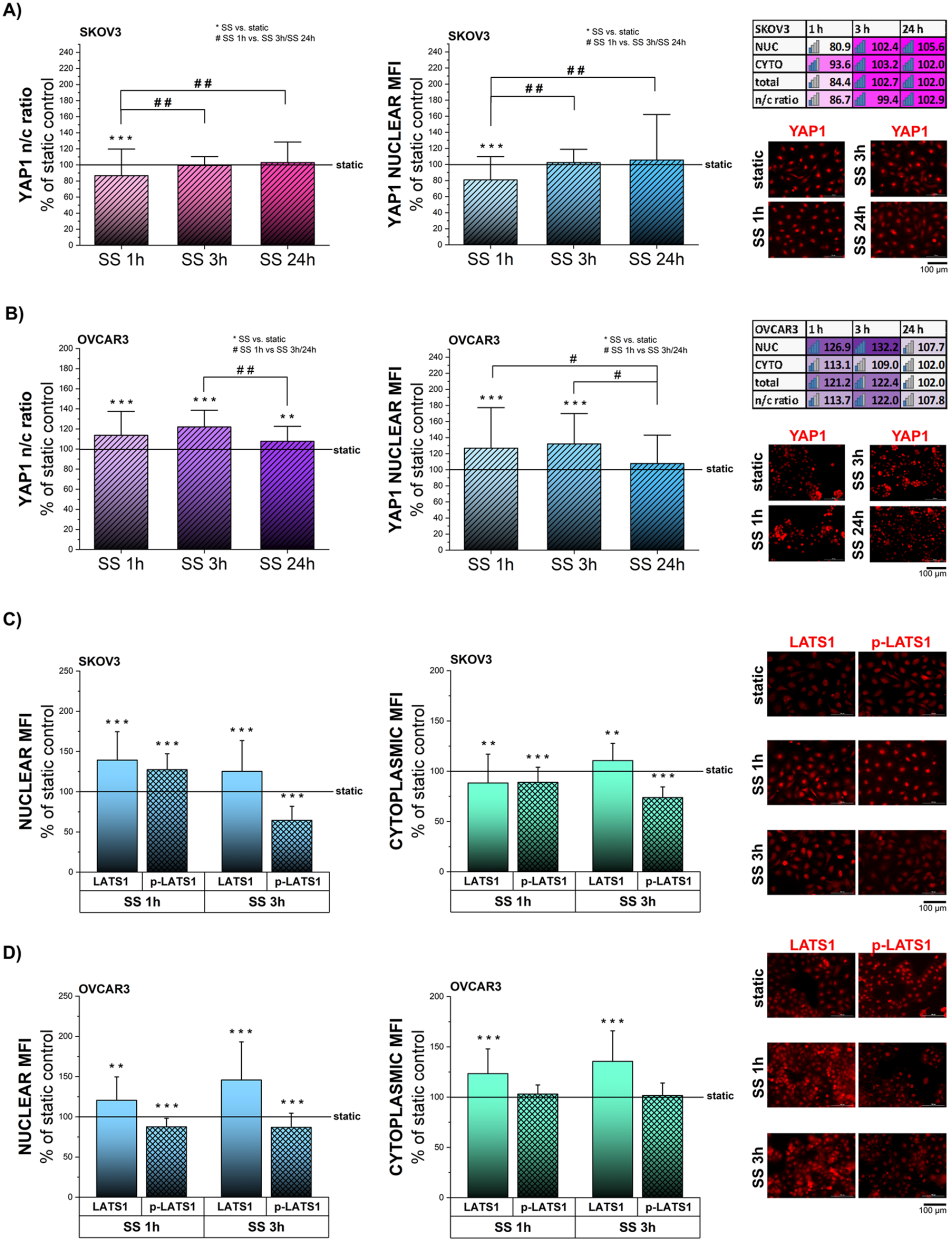
Shear stress stimulation in EOC: mechanosensitive regulation of YAP1. **A** + **B** Quantification of YAP1 n/c ratio and nuclear signal in cells exposed to 1 h, 3 h and 24 h of SS expressed as percentage of static control. Representative images show YAP1 depicted in red. Heatmap represents kinetics of changes in nuclear (NUC), cytoplasmic (CYTO), total YAP1 protein level and n/c ratio. Data expressed as percentage of static control. * depicts significant differences between SS and static control, # depicts significant differences between SS 1 h and SS 3 h, SS 24 h, respectively. **C** + **D** Quantification of LATS1 and p-LATS1 in nuclear and cytoplasmic compartment of the cells exposed to 1 h, 3 h SS, expressed as percentage of static control. Representative images show LATS1 and p-LATS1 depicted in red. All experiments were performed in at least 3 independent biological replicates. Statistical significance calculated with t-test and shown as */# *p* < 0.05, **/## *p* < 0.01, ***/### *p* < 0.001

As SREBP2 can contribute to the regulation of YAP1, the subcellular localization of the second mechanosensitive transcription factor was also monitored. YAP1 is a well-known sensor and mediator of physical signaling originating from the extracellular environment and its nuclear translocation is one of the best characterized mechanosensitive pathways [13, 14, 79]. In SKOV3, 1 h SS reduced YAP1 localization in the nuclear area in comparison to static controls (Fig. 6A). More prolonged mechanical stimulation supported a signal increase in the nuclear compartment as well as in n/c ratio in comparison to the quantification performed at 1 h. However, the signal variation was not significant in comparison to static controls (Fig. 6A). In agreement with the rapid formation of stress fibers observed in OVCAR3 (Fig. 3A), short term SS induced YAP1 translocation to the nucleus (1 h, 3 h Fig. 6B). After 24 h SS, YAP1 n/c ratios were maintained higher in comparison to static controls, albeit reduced in comparison to the values measured after 3 h SS (Fig. 6B). A similar trend could be followed in the quantification of the total nuclear YAP1 (Fig. 6B). To shed more light on the kinetics and signaling cascades supporting SS-induced YAP1 nuclear translocation, analysis of negative YAP1-regulator LATS1 and its activated phosphorylated form (p-LATS1) were performed, focusing on the nuclear and the cytoplasmic compartments (Fig. 6C-D). Nuclear p-LATS1 quantification revealed a complementary pattern to the movements described for YAP1. In SKOV3, after 1 h SS nuclear YAP1 signal was decreased and, at the same time, nuclear p-LATS1 increased. After 3 h SS, YAP1 nuclear localization increased, and this was allowed by decreased nuclear p-LATS1 (Fig. 6A and C). In OVCAR3, decreased p-LATS1 level at both timepoints (1 h and 3 h; Fig. 6D) aligned with increased YAP1 nuclear localization (Fig. 6B). Interestingly, nuclear p-LATS1 aligned more consistently with the YAP1 regulation in comparison to the cytoplasmic p-LATS1. Indeed, in OVCAR3 cells cytoplasmic p-LATS1 level was not altered in response to 1 h and 3 h SS, while YAP1 nuclear localization increased.

### Impact of PIEZO1 and calcium influx on the mechanosensitive regulation of SREPB2 and YAP1

**Fig. 7:**
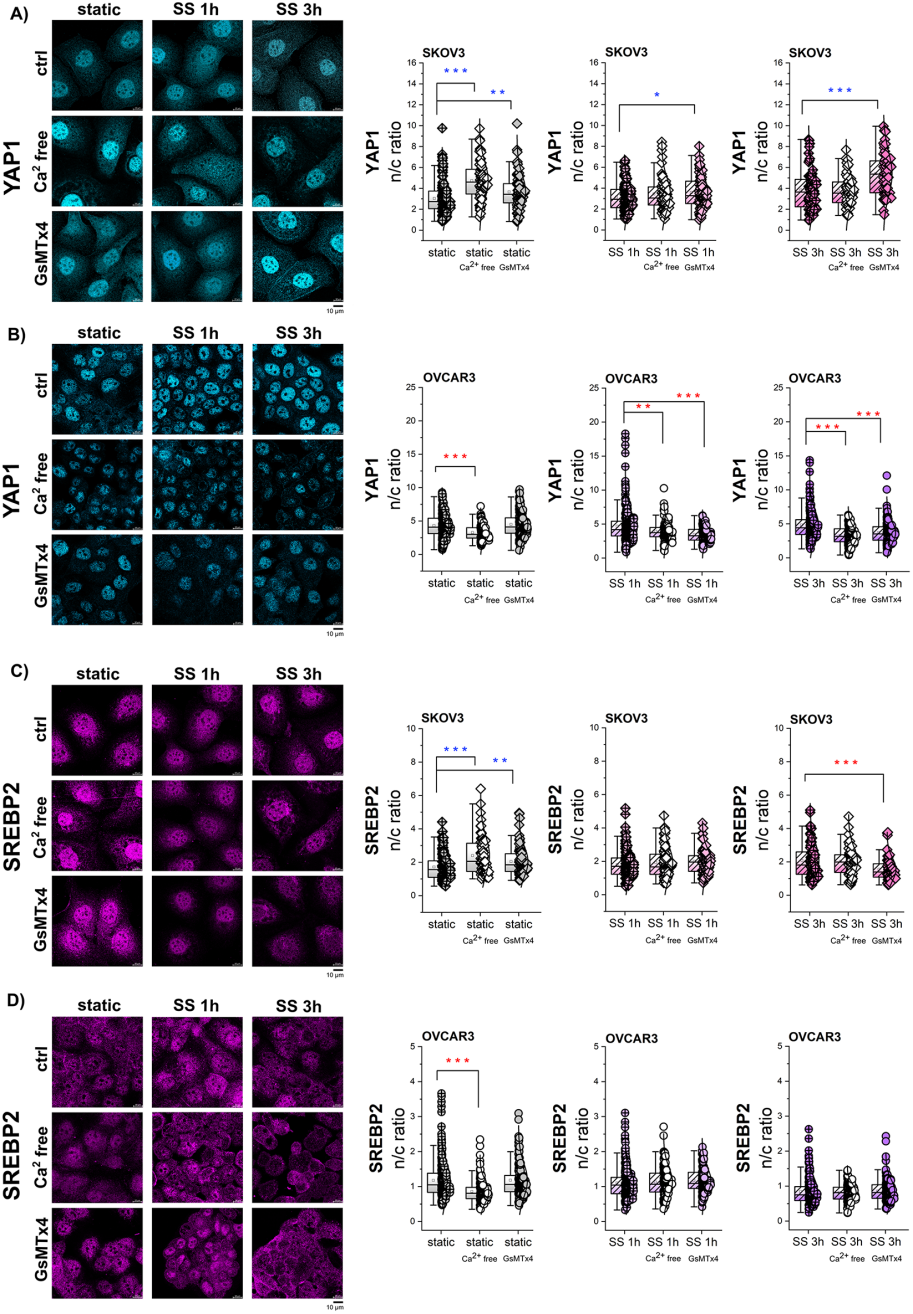
PIEZO1 and calcium-driven translocation of TFs. **A** + **B** Quantification of YAP1 n/c ratio in cells in static conditions or exposed to 1–3 h of SS with or without treatment (Ca^2+^ free, 1 µM GsMTx4). Representative images show YAP1 depicted in blue. * depicts significant differences between control and treatment. **C** + **D** Quantification of SREBP2 n/c ratio in cells in static conditions or exposed to 1–3 h of SS with or without treatment (Ca^2+^ free, 1 µM GsMTx4). Representative images show SREBP2 depicted in magenta. * depicts significant differences between control and treatment according to t-test (* *p* < 0.05, ** *p* < 0.01, *** *p* < 0.001). All experiments were performed in at least 3 independent biological replicates

To strengthen the postulated connection between mechanical stimulation and translocation of transcription factors YAP1 and SREBP2, further experiments were performed to explore the involvement of PIEZO1 channels activation. To isolate the contribution of the Ca^2+^ influx from the extracellular compartment, cells were exposed to shear stress in calcium-free extracellular medium. Additionally, to address more specifically the role of mechanogated ion channels, the chemical inhibitor of PIEZO1 channel GsMTx4 was included in the experimental layout [80]. In SKOV3 in static conditions, removal of extracellular Ca^2+^ increased YAP1 nuclear translocation (Fig. 7A). This was supported by the decreased nuclear level of p-LATS1 (Suppl. Figure S4 A) and constant appearance of the nuclear morphology (Suppl. Figure S4B). The application of GsMTx4 also triggered YAP1 nuclear translocation, thus retracing the response obtained in Ca^2+^ free medium (Fig. 7A, static). Additionally, in SKOV3, when mechanical stimulation was applied in the presence of GsMTx4, shear stress triggered a significant increase of YAP1 nuclear localization compared to control (Fig. 7A). For OVCAR3, absence of extracellular calcium decreased nuclear/cytoplasmic ratio of YAP1 in both static and shear stress conditions (Fig. 7B), however, it is worth noticing that nuclear morphology changed significantly with this experimental layout (Suppl. Figure S4B). Nevertheless, a similar response of decreased YAP1 n/c ratio was evoked in the presence of GsMTx4 and shear stress, supporting the importance of PIEZO1 activity in shear stress-induced YAP1 translocation (Fig. 7B). It has been reported, that calcium can either activate or inhibit the Hippo pathway and the final relationship between Ca^2+^ and Hippo pathway activity, namely YAP1 translocation, results from the interplay of several factors, such as actin cytoskeleton and intracellular cholesterol [81, 82]. To verify if the activation of the PIEZO1 alone could be sufficient to trigger the mobilization YAP1, or if physical deformation is necessary to observe the movement of the TF, chemical agonist YODA1 was applied. In this case no variations in actin intensity or area (cell morphology) could be observed (Suppl. Figure S5 A-B), as well as no YAP1 translocation (Suppl. Figure S5 C).

SREBP2 nuclear translocation aligned to YAP1 translocation pattern in static conditions in both cell lines. Calcium free conditions supported an increase in SREBP2 translocation to the nucleus in SKOV3, whereas a decrease in OVCAR3. The chemical inhibition of PIEZO1 had no effect on OVCAR3 and only sustained a slight increase in SREBP2 nuclear translocation in SKOV3. After 1 h shear stress no changes were observed in either cell line. After 3 h SS decrease in SREBP2 nuclear translocation was observed upon PIEZO1 inhibition only in SKOV3 (Fig. 7C-D).

### Impact of mevalonate pathway inhibition on the mechanosensitive regulation of YAP1

**Fig. 8:**
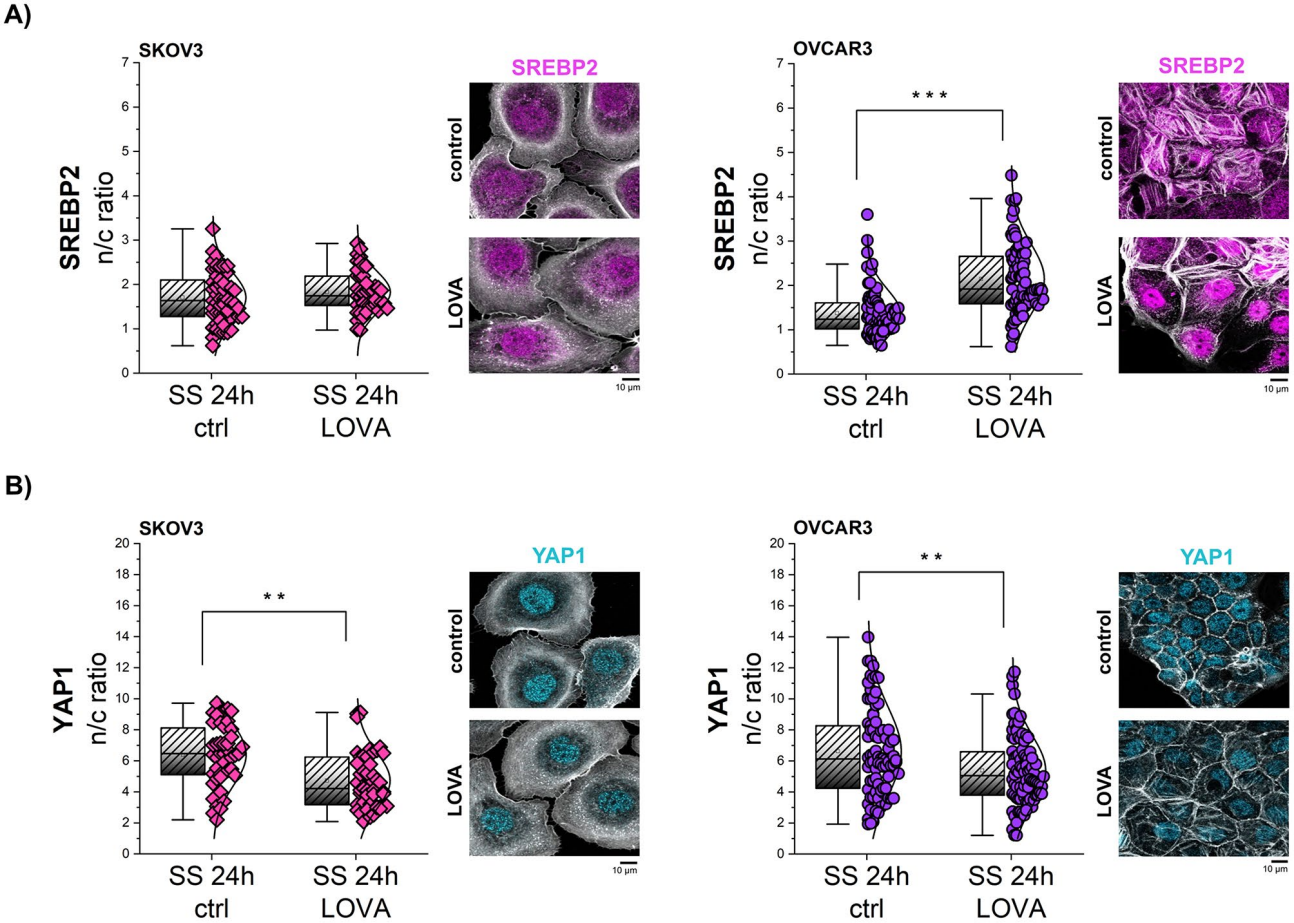
Mevalonate pathway inhibition regulates SS-induced YAP1 nuclear translocation. **(A)** Quantification of SREBP2 n/c ratio in cells exposed to 24 h SS with or without presence of LOVA 1 µM. **(B)** Quantification of YAP1 n/c ratio in cells exposed to 24 h SS with or without presence of LOVA 1 µM. Representative images show SREBP2 depicted in magenta, YAP1 depicted in blue, and actin depicted in white. All experiments were performed in at least 3 independent biological replicates. Statistical significance calculated with t-test and shown as * *p* < 0.05, ** *p* < 0.01, *** *p* < 0.001 between LOVA treatment and solvent control

Since both SREBP2 and YAP1 seemed to be responsive to shear stress in EOC cells, further experiments were performed to explore the connection between the two TFs. Indeed, it was previously shown that the mevalonate pathway affects subcellular localization of YAP1/TAZ and is one of the key drivers of its activity [22, 83]. To address possible regulation of YAP1 by SREBP2-driven mevalonate pathway in SKOV3 and OVCAR3 models, inhibitor of cholesterol synthesis lovastatin (LOVA) was applied concomitant to shear stress treatment. LOVA presence in combination with SS did not modify the translocation pattern of SREBP2 in SKOV3 when compared to SS alone (Fig. 8A). In parallel, LOVA induced an increase in LDLR expression at the PM in SKOV3, possibly supporting cholesterol uptake from the extracellular environment (Suppl. Figure S6 A). In OVCAR3 cells, application of SS protocol in presence of LOVA returned higher SREBP2 n/c ratios in comparison to SS alone (Fig. 8A). As far as YAP1 is concerned, the inhibition of cholesterol synthesis by LOVA diminished n/c signal ratios in both cell lines (Fig. 8B) underpinning the importance of active mevalonate pathway in YAP1 nuclear translocation upon mechanical stimuli. To additionally delve into mechanisms related to inhibition of cholesterol synthesis that might potentially influence YAP1 translocation, PIEZO1 expression and membrane properties were assessed in the two cell lines. Upon LOVA treatment, neither changes in PIEZO1 expression (Suppl. Figure S6B), nor membrane fluidity (Suppl. Figure S6 C) were observed in OVCAR3. For SKOV3, only a moderate decrease in membrane fluidity could be measured (Suppl. Figure S6 C).

### Shear stress stimulation in EOC: metabolome analysis

**Fig. 9:**
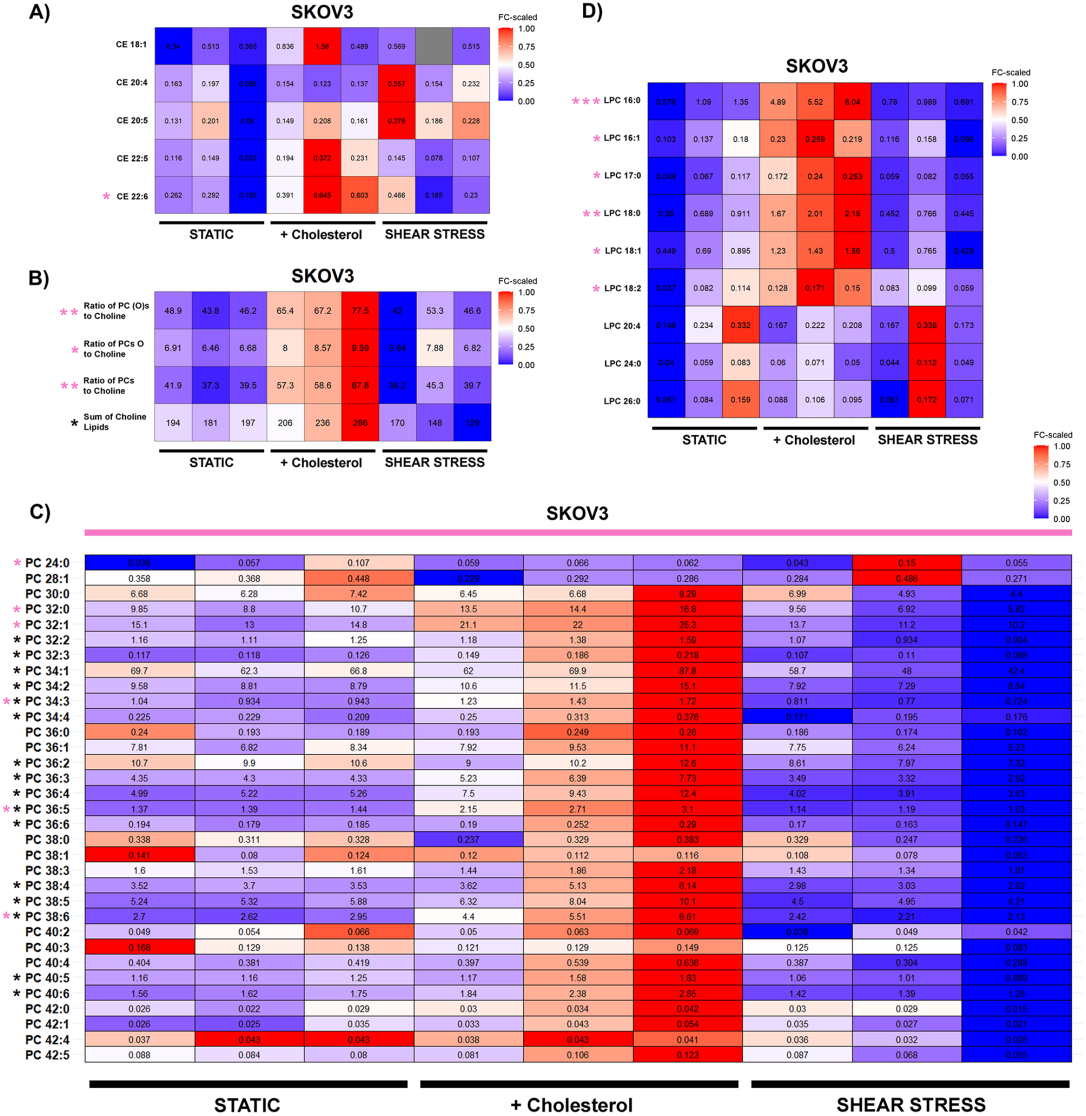
**A-D** Heatmaps showing graphical representation of metabolome data in SKOV3 cells comparing static controls, static controls treated with cholesterol 10 µg/ml (SKOV3) for 24 h, and cells exposed to SS for 24 h. If not otherwise specified, values represent metabolite concentrations [µM]. **(A)** cholesterol ester species (CE), **(B)** Ratios of PCs to choline, **(C)** phosphatidylcholine species (PC) **(D)** lyso-phosphatidylcholine species (LPC). Color coding from red-to-blue represents values from high-to-low. Gray entry represents measurement failure. All experiments were performed in at least 3 independent biological replicates. * black indicates statistical significance calculated with t-test between static control and 24 h shear stress, * pink indicates statistical significance calculated with t-test between treatment and control. Significance is shown as * *p* < 0.05, ** *p* < 0.01, *** *p* < 0.001

Even though the two cell lines differ in the lipid biosynthetic machinery [43] and total cholesterol levels (Fig. 1C), they showed the same trend in the regulation of lipid metabolism (i.e. SREBP2 translocation Fig. 4; cholesterol increase Fig. 5A). Therefore, to gain an insight into metabolic rewiring upon shear stress, we performed metabolomic analysis. As metabolomics datasets in relation to mechanotransduction are rare, experiments were performed including control treatments that aligned with the characteristic features of the two cell lines. SKOV3, relying more on the cholesterol uptake, were incubated with exogenous cholesterol (MβCD-cholesterol). OVCAR3 cells were treated with cholesterol biosynthesis inhibitor lovastatin (LOVA) as they rely more on the endogenous cholesterol synthesis. Viability of cells treated with SS and LOVA/Chol, respectively was not compromised (Suppl. Figure S7). Lipid quantification revealed increased cholesterol ester (CE) levels in SKOV3 cells treated with cholesterol (CE 22:6; Fig. 9A), as well as ratios of phosphatidylcholine (PC) to choline (Fig. 9B), levels of PC species (PC 24:0, PC 32:0, PC 32:1, PC 34:3, PC 36:5, PC 38:6; Fig. 9C), and lyso-phosphatidylcholine (LPC) levels (LPC 16:0, LPC 16:1, LPC 17:0, LPC 18:0, LPC 18:1, LPC 18:2; Fig. 9D) supporting the responsiveness of the system to the cholesterol treatment and providing reference for further interpretation of the metabolomics dataset. After 24 h shear stress application a slight increase in CE levels could be measured, albeit not significant (Fig. 9A). Further, the analysis was extended to include the behavior of PC and LPC. Following 24 h of shear stress, the total sum of choline lipids was significantly decreased, however, ratios of PC to choline were not affected (Fig. 9B). Aligned with overall decrease of choline lipids, levels of PC species (PC 32:2, PC 32:3, PC 34:1, PC 34:2, PC 34:3, PC 34:4, PC 36:2, PC 36:3; PC 36:4, PC 36:5, PC 36:6, PC 38:4, PC 38:5, PC 38:6, PC 40:5, PC 40:6) were significantly decreased after shear stress (Fig. 9C) with no changes of LPC levels (Fig. 9D).

**Fig. 10:**
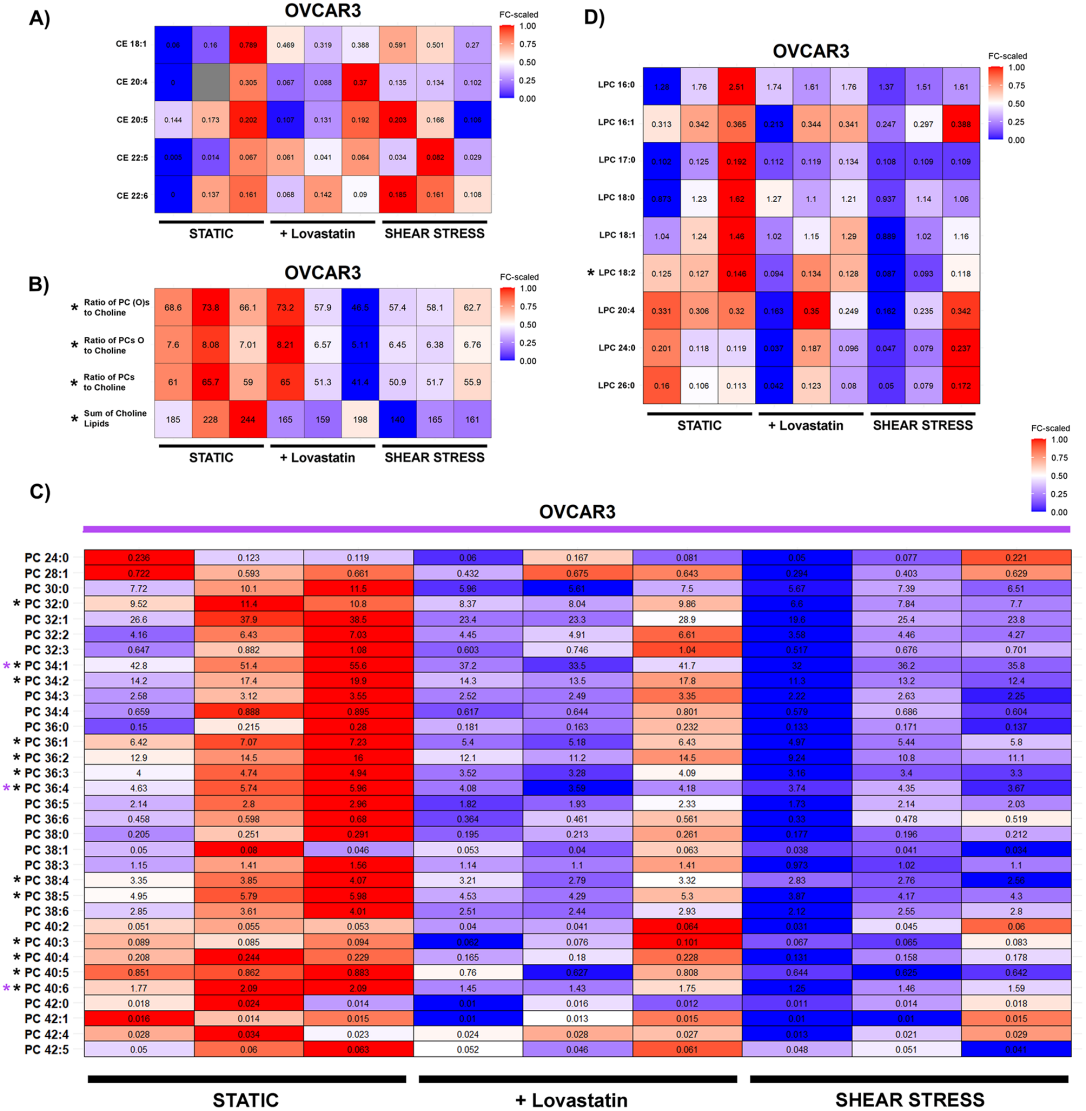
**A-D** Heatmaps showing graphical representation of metabolome data in OVCAR3 cells comparing static controls, static controls treated with lovastatin 1 µM for 24 h, and cells exposed to SS for 24 h. If not otherwise specified, values represent metabolite concentrations [µM] **(A)** cholesterol ester species (CE), **(B)** Ratios of PCs to choline, **(C)** phosphatidylcholine species (PC) **(D)** lyso-phosphatidylcholine species (LPC). Color coding from red-to-blue represents values from high-to-low. Gray entry represents measurement failure. All experiments were performed in at least 3 independent biological replicates. * black indicates statistical significance calculated with t-test between static control and 24 h shear stress, * violet indicates statistical significance calculated with t-test between treatment and control. Significance is shown as * *p* < 0.05, ** *p* < 0.01, *** *p* < 0.001

In OVCAR3 cells, LOVA treatment had no effect on CE levels (Fig. 10A) and slightly decreased ratios of phosphatidylcholine (PC) to choline, albeit not statistically significant (Fig. 10B). However, the signature seemed clearer for the PC species, where a significant decrease of PC 34:1, PC 36:4, PC 40:6 could be observed (Fig. 10C). LPC levels were not affected by LOVA treatment (Fig. 10D). 24 h SS application did not alter CE levels in OVCAR3 (Fig. 10A), but on the contrary to SKOV3 cells, shear stress significantly decreased ratios of phosphatidylcholine (PC) to choline (Fig. 10B), with concomitant decrease of PC levels (PC 32:0, PC 34:1, PC 34:2, PC 36:1, PC 36:2, PC 36:3, PC 36:4, PC 38:4, PC 38:5, PC 40:3, PC 40:4, PC 40:5, PC 40:6; Fig. 10C). LPC levels were not widely regulated by SS, only LPC 18:2 was significantly decreased (Fig. 10D). Overall, coherent response of SKOV3 and OVCAR3 to SS seems to agree with the microscopy-based experiments (Fig. 4), thus suggesting that after exposure to shear stress SKOV3 and OVCAR3 regulate the lipid metabolism in the same direction.

### Shear stress stimulation in EOC: effect on the chemosensitivity to BOLD-100

**Fig. 11:**
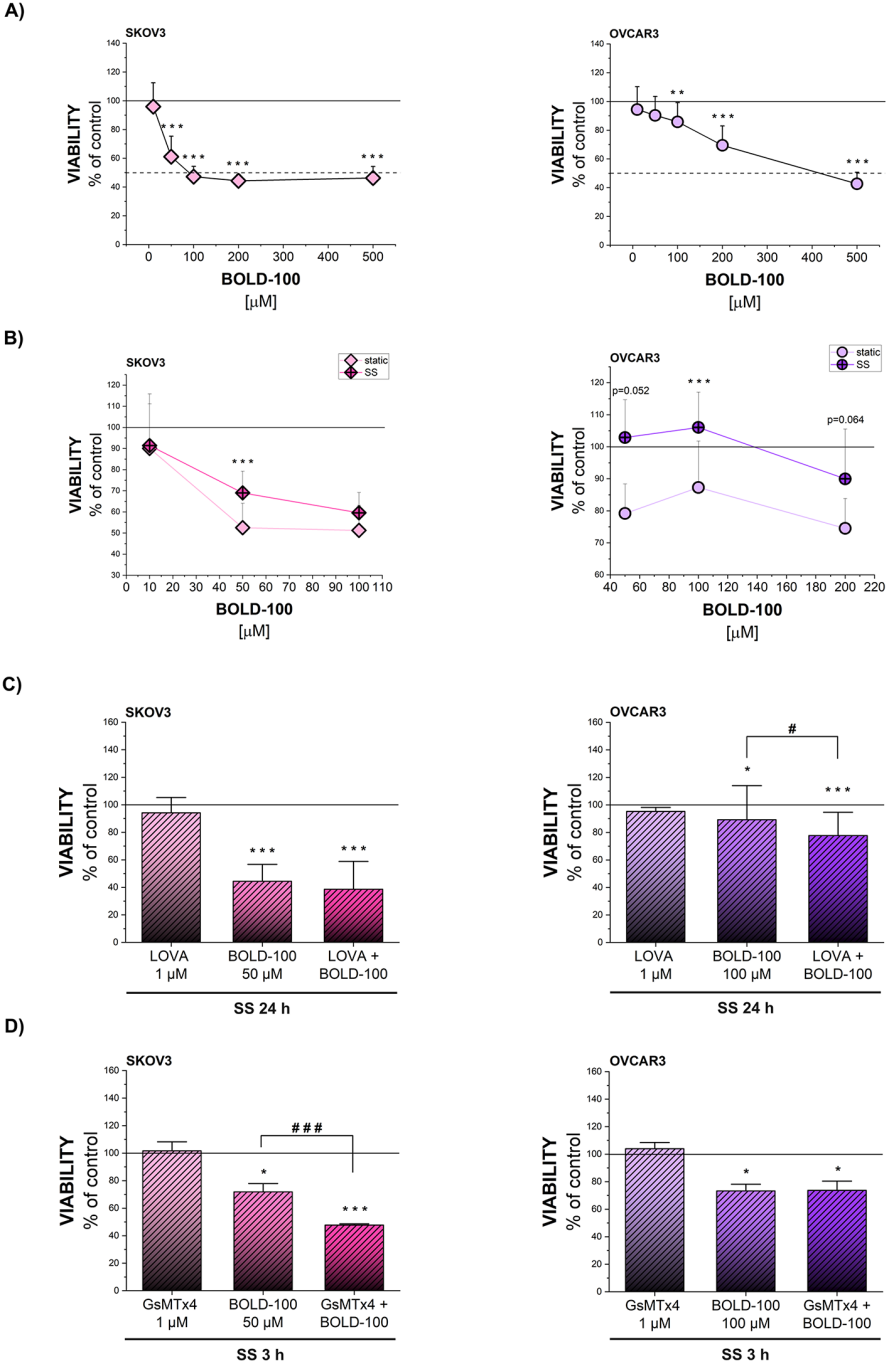
Effect of SS on chemoresistance of OC cells to BOLD-100. **(A)** Viability of cells treated with BOLD-100 in concentration range 10–500 µM for 48 h measured by crystal violet assay. Results expressed as percentage of solvent control. **(B)** Viability of cells treated with BOLD-100 in concentration range 10–100 µM for 48 h for SKOV3 and 50–200 µM for 48 h for OVCAR3 with or without preconditioning with 24 h SS measured by crystal violet assay. Results expressed as percentage of solvent control for both static and SS conditions. * depicts significant difference between static and SS conditions. **(C)** Viability of cells exposed to 24 h SS with or without presence of LOVA (1 µM) and afterwards treated with BOLD-100 in concentration 50 µM (SKOV3), 100 µM (OVCAR3) for 48 h, measured by crystal violet assay. * depicts significant difference between solvent control and treatments, # depicts significant difference between BOLD-100 treatment and combination LOVA and BOLD-100. **(D)** Viability of cells exposed to 3 h SS with or without presence of GsMTx4 (1 µM) and afterwards treated with BOLD-100 in concentration 50 µM (SKOV3), 100 µM (OVCAR3) for 48 h, measured by crystal violet assay. * depicts significant difference between solvent control and treatments, # depicts significant difference between BOLD-100 treatment and combination GsMTx4 and BOLD-100. Statistical significance was calculated with t-test and multiple comparisons by one-way ANOVA test and shown as */# *p* < 0.05, **/## *p* < 0.01, ***/### *p* < 0.001

As shear stress appeared to be able to modulate lipid metabolism and uptake retracing pathological mechanism relevant for tumor growth, additional experiments were designed to verify potential interactions with chemotherapeutic agents. Recently, lipid metabolism has been reported to be a modulator of the anticancer activity of BOLD-100, which is currently in Phase II of clinical evaluation. Despite being a promising compound, mechanistic studies on colon cancer cells resistant to the ruthenium-containing drug evidenced an increase in the expression of proteins related to the cholesterol biosynthetic pathway, inferring for a link between lipid metabolism and the chemo-resistant phenotype [84]. Taking this as a starting point, the compound was chosen to correlate the lipid metabolism profile in SKOV3 and OVCAR3 to drug-responsiveness. Aligning with the cholesterol content data, cytotoxicity assay revealed that SKOV3 cells were more sensitive to BOLD-100 than OVCAR3 (EC 40.2 µM vs. 271.2 µM, respectively; Fig. 11A). Cell death in OVCAR3 was related to the activation of the Caspase3/7 (Suppl. Figure S8A). In parallel to the modulation of lipid metabolism, exposure to shear stress 24 h (SS-preconditioning) decreased the sensitivity of SKOV3 and OVCAR3 to BOLD-100 (Fig. 11B). Postulating an increase in cholesterol biosynthesis via mevalonate pathway as responsible for the reduced response to the treatment, experiments were performed in presence of LOVA. Inhibition of cholesterol biosynthesis decreased the viability of BOLD-100 treated cells compared to BOLD-100 alone both in SS and static conditions in OVCAR3 cells, nevertheless had limited effect in SKOV3 cells (Fig. 11C, Suppl. Figure S8B). To further strengthen the evidence that the postulated increased chemoresistance could stem from mechanical stimuli, PIEZO1 inhibitor GsMTx4 was applied during 3 h SS-preconditioning. Following 48 h of BOLD-100 treatment, viability of SKOV3 cells co-treated with GsMTx4 and BOLD-100 was significantly lower than control as well as than BOLD-100 alone (Fig. 11D). In OVCAR3, BOLD-100 toxicity was not modulated by the presence of GsMTx4 (Fig. 11D).

## Discussion

Ovarian cancer is a highly aggressive gynecologic malignancy and remains one of the leading causes of cancer-related death in women worldwide [85]. Despite advances in treatment, the high mortality rate associated with ovarian cancer is largely due to the development of chemoresistance, which limits the efficacy of standard therapies such as chemotherapy [86–88]. Recent research has highlighted the critical role of lipid metabolism in ovarian cancer, demonstrating that the altered lipid processing not only supports cancer cell growth and survival, but also contributes to the development of resistance to treatments [89]. In addition, metastatic ovarian cancer cells avidly adhere to the omental fat pad lining the intestine and the peritoneal cavity is the most common site of metastatic colonization [78]. Ovarian cancer cells preferentially migrate and form metastases in fat-rich environments [75–78], highlighting the need to understand the interplay between lipid metabolism and ovarian cancer phenotypes. Elucidation of such mechanisms is seen of central importance for the development of novel strategies to improve therapeutic outcomes for ovarian cancer patients [89, 90].

In addition to metabolic features, recent studies have brought to light the importance of physical cues in shaping cancer aggressiveness. Fluid shear stress can induce chemoresistance to platinum-based drugs in ovarian cancer cells, namely promoting the acquisition of cancer stem cell markers and epithelial-to-mesenchymal transition, and ultimately leading to increased resistance to cisplatin and carboplatin [11, 91]. This resistance is associated with changes in mitochondrial function and increased EGFR signaling [42, 91]. While SS confers resistance to platinum-based drugs, it seems to affect less the sensitivity to doxorubicin [91]. Novel approaches to overcome SS-induced chemoresistance include photodynamic priming using benzoporphyrin derivative or ALA-PpIX, and EGFR-targeted photoimmunotherapy, which show promise in enhancing platinum drug efficacy in presence of SS [42, 91].

The present work further contributes to elucidating how mechanical cues could relate to chemoresistance. Particularly, it focused on the relationship between mechanical stimulation and altered endogenous lipid metabolism. In this context, SKOV3 and OVCAR3 cells used in this study differ significantly in the expression of lipid biosynthesis machinery [43]. In line, SKOV3 are equipped with a thin, cholesterol-poor membrane (Fig. 1B and C) which exhibits higher fluidity (Fig. 1D). Additionally, the membrane of SKOV3 cells presents more abundant membrane folds and grooves (Fig. 1E and F), which is consistent with higher expression of the caveolae-associated protein CAV-1 (Fig. 1G). These structural features, seem to serve as membrane “reservoirs” possibly providing flexibility necessary for highly motile cell types such as SKOV3 [92] and likely facilitating cell spreading in response to physical stimulation (Fig. 3A [43, 64, 93]). Mechanical stress has been previously described to induce rapid disassembly and flattening of caveolae, which buffers membrane tension and protects cells from damage [63, 94]. Further connecting membrane morphology to functional adaption to SS, OVCAR3 cells have a thick and rigid membrane with high cholesterol content (Fig. 1B-D) and maintained constant area upon 3 h shear stress application (Fig. 3A). This stability was accompanied by actin stress fiber formation (Fig. 3A), which is in accordance with previous findings [10].

Among the key molecules at the PM sensing external mechanical stimuli is mechanosensitive ion channel PIEZO1 [95]. Based on different PM properties between SKOV3 and OVCAR3 cells, PIEZO1 expression and activity were evaluated. OVCAR3 returned higher increase of [Ca^2+^]_i_ when stimulated with PIEZO1 agonist YODA1 (Fig. 2A-C), which is in accordance with previously described higher expression of proteins related to calcium management [43]. Additionally, in OVCAR3 PIEZO1 detection at the PM rapidly decreased after mechanical stimulation (Fig. 2D). PIEZO1 physically interacts with cholesterol in the PM [96, 97] and recent studies have revealed the crucial role of cholesterol in regulating its function. PIEZO1 forms nanometric clusters in the plasma membrane, which are sensitive to membrane manipulation and depend on cholesterol domains for coordinated activity [18, 96]. These findings suggest that cholesterol plays a protective role in PIEZO1 function by regulating its organization and activity, potentially preventing excessive Ca^2+^ influx [98]. On this basis, it is possible that cholesterol increase at the PM (Fig. 5A) could mask PIEZO1 protein, which would become less accessible for immunofluorescence detection. This phenomenon was previously described for another PM-embedded protein, namely Caveolin-1 [99] and also experiments performed with SKOV3 and OVCAR3 show decrease of Caveolin-1 signal intensity (Suppl. Figure S9 A) parallel to SS-induced cholesterol accumulation (Fig. 5A). To support this line of interpretation, treatment with cholesterol synthesis inhibitor LOVA increased Caveolin-1 detection in OVCAR3 cells (Suppl. Figure S9B).

Further following inward signaling stemming from membrane deformation and activation of PIEZO1 channels, the involvement of the cytoskeleton and related proteins was evaluated. In addition to morphological changes, the formation of actin stress fibers provides molecular prerequisites for the translocation of transcription factors [100]. Lipid metabolism is under the transcriptional control of the SREBP family of proteins. Reprogramming of lipid metabolism in cancer cells has emerged as one of the metabolic hallmarks of cancer [101] that further supports the others [102]. In particular, SREBP2 drives the biosynthesis and uptake of cholesterol [103, 104] and these metabolic abnormalities were recently identified as unfavorable markers of endometrial cancer [105]. Additionally, it has been observed that localization and activity of SREBP1 and SREBP2 can be modulated by external mechanical forces [36, 106]. The shear stress-induced translocation pattern of SREBP2 observed in this study (Fig. 4) sustains the existence of different routes or kinetic of activation supporting the nuclear increase of the TF for SKOV3 and OVCAR3 cells. Provided with the same physical challenge, OVCAR3, where actin stress fibers could be observed (Fig. 3A), returned slightly faster increase of TF in comparison to SKOV3 (Fig. 4F), which was retraced in the cholesterol accumulation (Fig. 5A). Supportive of a causal relationship between SREBP2 translocation and membrane cholesterol accumulation, the nuclear/cytoplasmic ratio of SREBP2 was decreased compared to static controls in both cell lines after 24 h SS (Fig. 4D). This is likely related to the fact that SREBP2 activity is under the control of a feedback regulatory system, that senses intracellular cholesterol levels and modulates SREBP2 processing and thus the transcription of genes encoding for lipogenic enzymes [73]. This interpretation would agree with the kinetic of cholesterol detection observed after shear stress (Fig. 5A) and also with the metabolome data (Figs. 9 and 10). Similar results, namely increased nuclear localization of SREBP2 upon shear stress, were obtained by Traore et al. in renal cells, but with a longer stimulus duration (48 h) [107].

Delving deeper in the different response of SKOV3 and OVCAR3, SREBP2 not only induces cholesterol biosynthesis, but also its uptake from the extracellular environment via LDLR [104]. In OVCAR3, LDLR signal remained stable upon shear stress (Fig. 5C) regardless of cells’ capacity to accumulate cholesterol (Fig. 5A). In turn, SKOV3 revealed significant increase in membrane expression of LDLR after 3 h of shear stress. This underpins that SKOV3 cells could privilege extracellular cholesterol uptake, as supported by the experiments performed in lipid-depleted conditions (Fig. 5D). On the other hand, OVCAR3 cells seem to rely more on endogenous synthesis. This interpretation agrees with the incapacity of OVCAR3 to increase cholesterol upon shear stress in presence of the inhibitor lovastatin (Fig. 5B). Moreover, this is consistent with higher basal level of the mevalonate pathway intermediate farnesyl in this cell line (Suppl. Figure S10), as well as with previously performed untargeted proteomic analysis [43] that described higher expression of lipid synthesis-related proteins in OVCAR3 in comparison to SKOV3. Overall, this supports the view that ovarian cancer cells may use different strategies to increase cholesterol levels, where the latter was previously reported to support chemoresistance [30]. This observation is also highly relevant for ovarian cancer pathophysiology, as tumor cells prefer to metastasize in lipid-rich areas [76].

Recent studies have revealed a complex relationship between SREBP2 and YAP1/TAZ activity, highlighting their interrelated roles in metabolism and growth regulation. The SREBP2-mevalonate pathway has been shown to promote YAP/TAZ activity [22] and to maintain YAP/TAZ nuclear localization by providing essential precursors for protein prenylation and RHO membrane localization [108]. Consistent with this, the inhibition of endogenous cholesterol biosynthesis by LOVA abolished YAP1 translocation in OC cells (Fig. 8B). This effect seemed to be independent on SREBP2 nuclear translocation (Fig. 8A) implying the active mevalonate pathway to be crucial for YAP1 translocation, as previously described in literature [22]. Additionally, it is worth noticing that incubation with LOVA also modified CAV-1 signal in OVCAR3 cells (Suppl. Figure S9B), thus suggesting for the inhibitor the capacity to modulate elements of the cell mechanosensory apparatus [109].

In ovarian cancer, altered lipid metabolism has been shown to support cancer cell proliferation, tumor progression, metastasis and resistance to anticancer drugs [110]. In particular, dysregulated cholesterol metabolism has been proposed as a metabolic hallmark in ovarian cancer, among others [111]. In this context, SREBP2 activity has been associated with increased chemoresistance of ovarian cancer cells towards platinum-based drugs [30]. The experimental ruthenium-containing drug BOLD-100 was used to investigate the relationship between shear stress-induced alterations in lipid metabolism and chemoresistance of OC cells. Recently, the cytotoxicity of BOLD-100 treatment has been shown to be largely influenced by endogenous lipid metabolism. In the same study, according to the KEGG database, gene sets associated with lipid metabolism were enriched in BOLD-100-resistant HCT116 cells, namely “LINOLEIC_ACID_METABOLISM” and “STEROID-BIOSYNTHESIS”, and in the Reactome database, “CHOLESTEROL-BIOSYNTHESIS” was identified as the top fourth highest upregulated gene set [84]. This is in line with the present study supporting lipid metabolism as a driver of chemoresistance in ovarian cancer cells: OVCAR3 cells with higher basal levels of cholesterol (Fig. 1C) show greater resistance toward BOLD-100 than SKOV3 (Fig. 11A). Furthermore, shear stress-induced cholesterol biosynthesis/uptake resulted in decreased sensitivity to BOLD-100 (Fig. 11B) and this was dependent on endogenous cholesterol biosynthesis in OVCAR3 (Fig. 11C). LOVA treatment did not alter sensitivity to BOLD-100 in SKOV3, which can be explained by greater dependence of this cell line on uptake of extracellular cholesterol, as shown in Fig. 5B and C. Interestingly, chemical inhibition of PIEZO1 channel activation by shear stress led to decreased nuclear localization of SREBP2 only in SKOV3 (Fig. 7C). Along this line, GsMTx4 treatment increased sensitivity to BOLD-100 in SS preconditioned SKOV3 cells and no change was observed in OVCAR3 (Fig. 11D), further sustaining the view that SKOV3 and OVCAR3 could take advantage of different strategies to modulate metabolic pathways and resistance. Recent studies propose that statins may have potential benefits in ovarian cancer therapy [89]. Epidemiologic evidence suggests that statin use, particularly lipophilic statins, is associated with reduced ovarian cancer risk and improved survival [112]. Although the mechanisms involved are poorly understood, statin use after diagnosis of ovarian cancer is associated with increased life expectancy [113], and considering the high mechanical stimulation experienced by the ovarian cancer cells in the abdominal cavity [8, 114], these clinical observations appear coherent with the experimental data obtained in the present study.

## Conclusions

Taken together, our data support the presence of a link between mechanical stimulation of ovarian cancer cells and rewiring of lipid metabolism, ultimately leading to altered sensitivity to anticancer drugs like BOLD-100. Furthermore, even with the clear limitations of cell culture models, the data in this study supports a role for the mevalonate pathway in mediating adaptation to the biophysical tumor microenvironment of ovarian cancer. The tumor cell heterogeneity, represented in this study by SKOV3 and OVCAR3 cell lines, seems to be a key element for the plasticity necessary to respond to external cues via different routes while still being able to achieve a similar phenotype, namely fine-tuning of lipid metabolism and resistance to treatment. Altogether, this study sustains the existence of mechanical-driven pathways that can prime tumor aggressiveness and open new perspectives for further exploration for possible Achilles’ heel of ovarian cancer cells.

## Electronic supplementary material

Below is the link to the electronic supplementary material.

Supplementary Material 1

## Data Availability

Data will be made available from the corresponding author at reasonable request.
