# Supplementary material for: Mechanical cues rewire lipid metabolism and support chemoresistance in epithelial ovarian cancer cell lines OVCAR3 and SKOV3
Source: Cell Commun Signal. 2025 Apr 22;23:193. doi: 10.1186/s12964-025-02144-9 (PMC12016438; doi:10.1186/s12964-025-02144-9)
Supplement: Supplementary file 1 — Supplementary Material 1 [file 12964_2025_2144_MOESM1_ESM.pdf]

SUPPLEMENTARY MATERIALS

Mechanical cues rewire lipid metabolism and support chemoresistance in epithelial ovarian cancer cell lines OVCAR3 and SKOV3

Martina Karasová [1], Maximilian Jobst [1,2], Denise Framke [1], Janice Bergen [1,2], Samuel Meier-Menches [3,4], Bernhard Keppler [4], Gunda Koellensperger [3], Jürgen Zanghellini [3], Christopher Gerner [3], Giorgia Del Favero [1*]

1 Department of Food Chemistry and Toxicology, Faculty of Chemistry, University of Vienna, Währinger Str. 38-40, 1090 Vienna, Austria

2 Doctoral School of Chemistry (DoSChem), Faculty of Chemistry, University of Vienna, Währinger Str. 42, 1090 Vienna, Austria

3 Department of Analytical Chemistry, Faculty of Chemistry, University of Vienna, Währinger Str. 38, 1090 Vienna, Austria

4 Department of Inorganic Chemistry, Faculty of Chemistry, University of Vienna, Währinger Str. 42, 1090 Vienna, Austria

* Correspondence

Supplementary figure 1

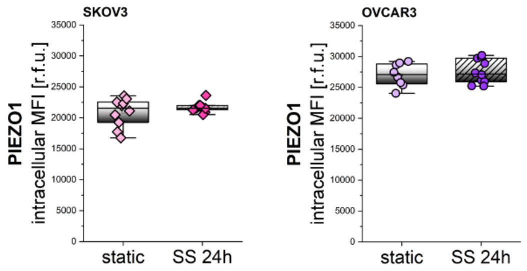

Suppl. Fig. 1 *Intracellular expression of Piezo1 in cells exposed to 24 h of SS. Images were acquired with Lionheart FX automated microscope. Data represents n>9 optical fields. All experiments were performed in at least 3 independent biological replicates. Statistical significance calculated with t-test and shown as * p < 0.05, ** p < 0.01, *** p< 0.001.*

Supplementary figure 2

A)

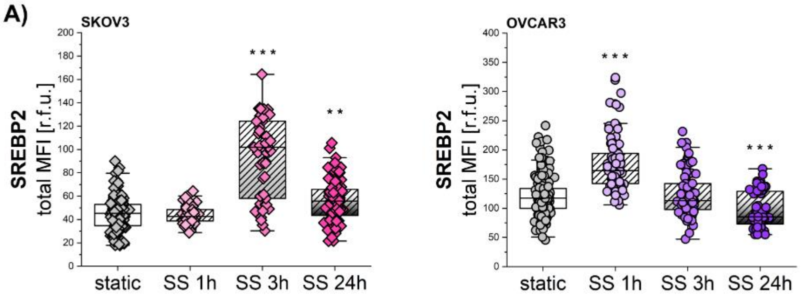

B)

SKOV3	1h	3h	24h
NUC	93.80	217.88	112.01
CYTO	93.19	169.62	152.99
total	93.54	205.04	122.97
n/c ratio	103.09	125.43	74.35

OVCAR3	1h	3h	24h
NUC	136.50	100.45	76.45
CYTO	135.97	125.63	101.64
total	136.25	109.97	86.58
n/c ratio	103.08	68.63	67.27

Suppl. Fig. 2 A) *SREBP2 total protein level in cells exposed to 1 h, 3 h and 24 h of SS and in respective static controls. Images were acquired with LSM Zeiss 710 equipped with ELYRA PS.1 system and data represent single cells (n>50)* ***B)*** *Heatmaps showing the kinetics of SREBP2 nuclear, cytoplasmic and total expression level, as well as nuclear/cytoplasmic ratio (n/c ratio). Data expressed as percentage of static control. All experiments were performed in at least 3 independent biological replicates. Statistical significance calculated with t-test and shown as * p < 0.05, ** p < 0.01, *** p< 0.001.*

Supplementary figure 3

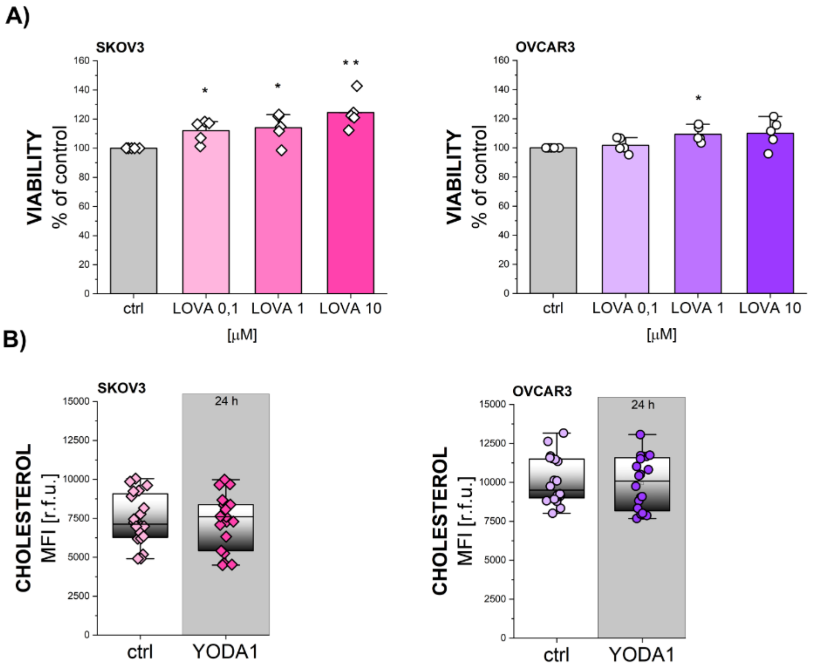

Suppl. Fig. 3 A) *Viability of cells treated by LOVA (0.1 μM, 1 μM and 10 μM) in static conditions. Data are expressed as percentage of solvent control.* ***B)*** *Quantification of cholesterol in cells exposed to YODA1 (1 μM) for 24 h. Images were acquired with Lionheart FX automated microscope. Data represents n>15 optical fields. All experiments were performed in at least 3 independent biological replicates. Statistical significance calculated with t-test and shown as * $p < 0.05$, ** $p < 0.01$, *** $p < 0.001$.*

Supplementary figure 4

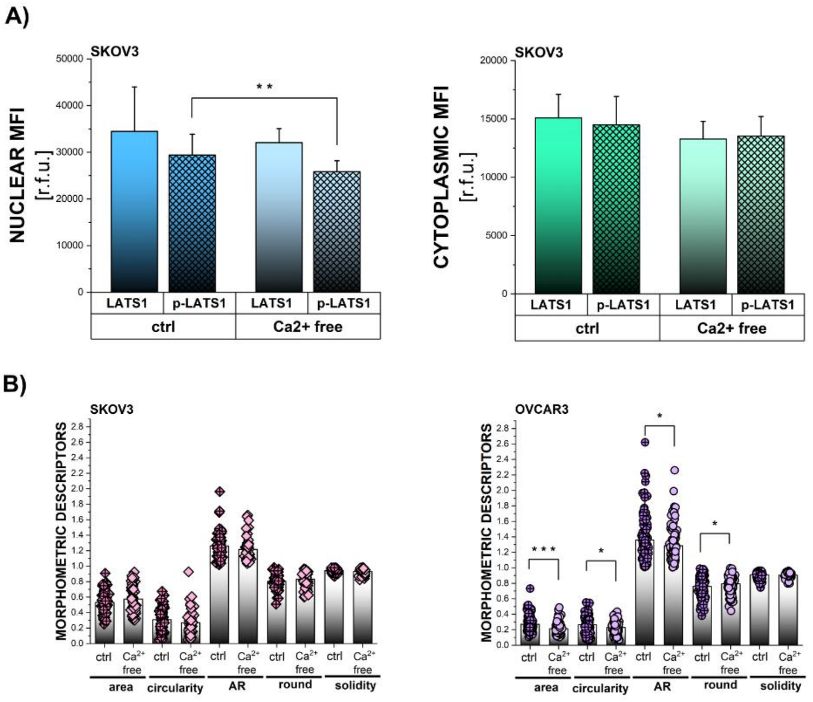

Suppl. Fig. 4. A) *Quantification of LATS1 and p-LATS1 in nuclear and cytoplasmic compartment in static conditions with or without Ca^{2+} ions (3 h). Images were acquired with Lionheart FX automated microscope.* ***B)*** *Morphometric analysis of cell nuclei exposed to control and Ca^{2+} free conditions (3 h). Image analysis performed with ImageJ software. All experiments were performed in at least 3 independent biological replicates. Statistical significance calculated with t-test and shown as * $p < 0.05$, ** $p < 0.01$, *** $p< 0.001$.*

Supplementary figure 5

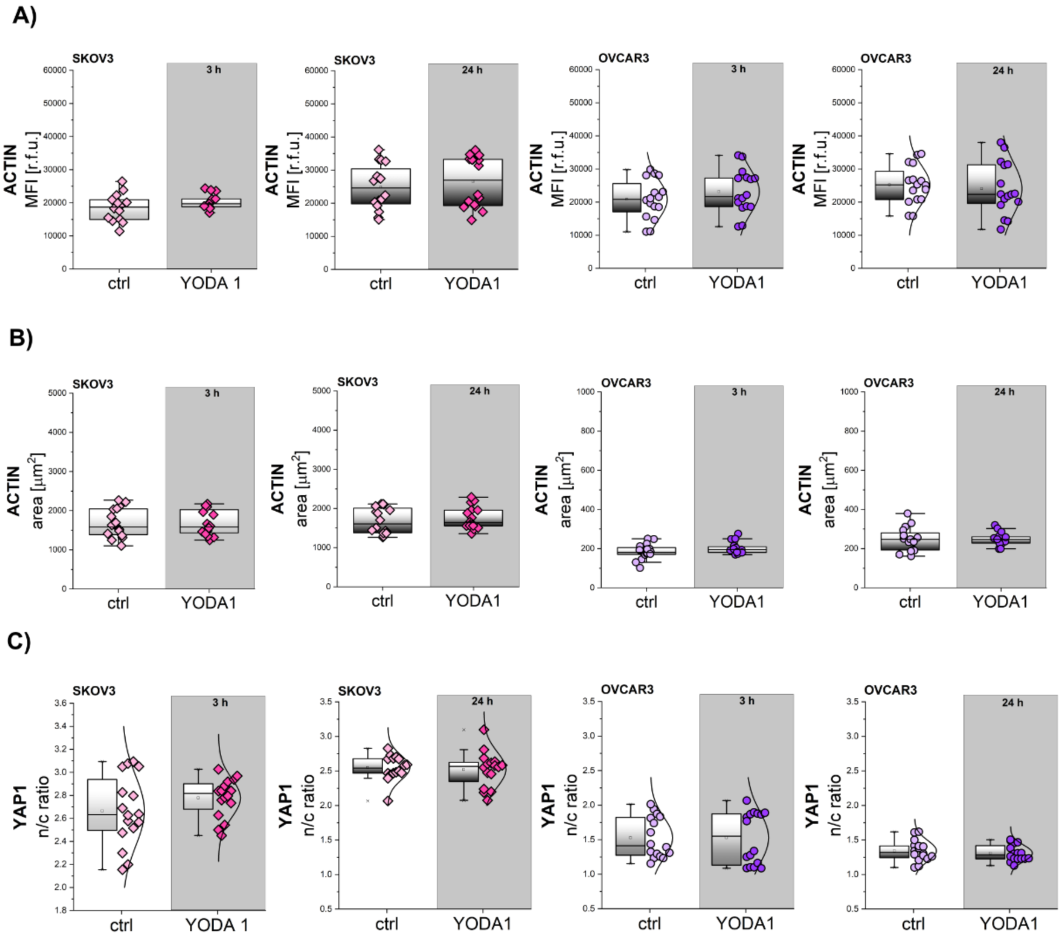

Suppl. Fig. 5 A) *Actin cytoskeleton intensity (r.f.u.) in cells exposed to YODA1 (1 μM) for 3 h and 24 h.* ***B)*** *Actin cytoskeleton area (μm^2) in cells exposed to YODA1 (1 μM) for 3 h and 24 h* ***C)*** *YAP1 subcellular localization (n/c ratio) in cells exposed to YODA1 (1 μM) for 3 h and 24 h. Images were acquired with Lionheart FX automated microscope. Data represents n>15 optical fields. All experiments were performed in at least 3 independent biological replicates. Statistical significance calculated with t-test and shown as * $p < 0.05$, ** $p < 0.01$, *** $p < 0.001$.*

Supplementary figure 6

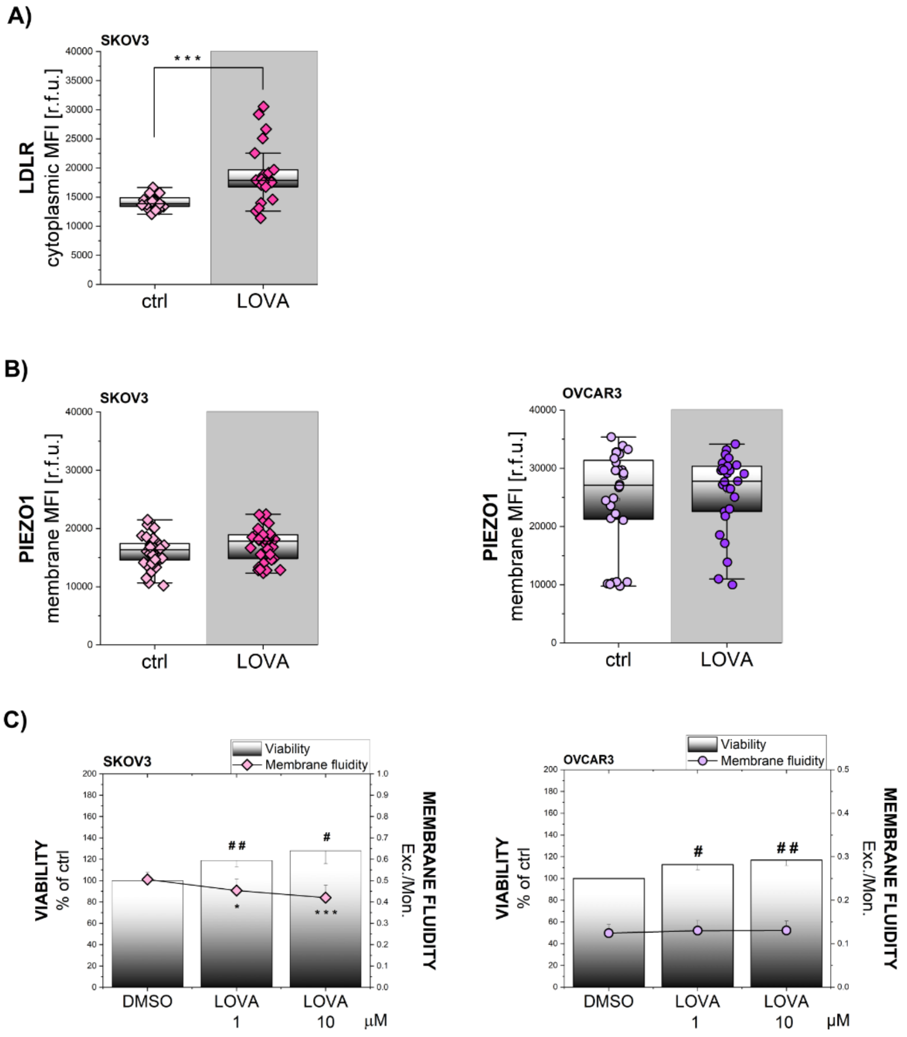

Suppl. Fig. 6 A) *Quantification of LDLR expression at the plasma membrane in cells exposed to LOVA (1 μM) for 24 h. Images were acquired with Lionheart FX automated microscope. Data represents n>15 optical fields.* ***B****) Quantification of PIEZO1 expression at the plasma membrane in cells exposed to LOVA (1 μM) for 24 h. Images were acquired with Lionheart FX automated microscope. Data represents n>15 optical fields.* ***C)*** *Membrane fluidity (PDA excimers/monomers ratio) and viability (% of solvent control) in cells treated with LOVA (1 μM and 10 μM) for 24 h. All experiments were performed in at least 3 independent biological replicates. Statistical significance calculated by t-test and shown as */# p < 0.05, **/## p < 0.01, ***/### p< 0.001.*

Supplementary figure 7

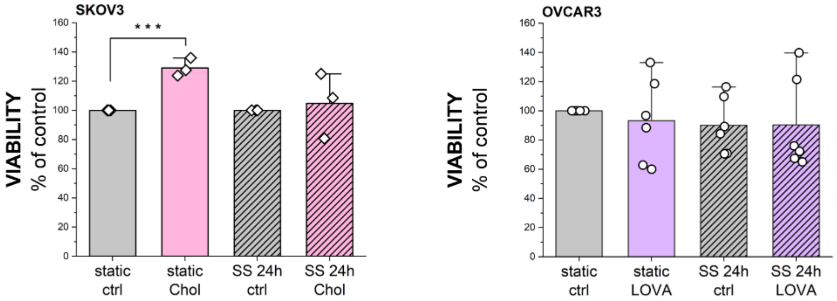

Suppl. Fig. 7 *Viability of cells treated with SS with or without treatment with LOVA (1 µM), Chol (10 µg/ml) for 24 h. Data are expressed as percentage of solvent control. All experiments were performed in at least 3 independent biological replicates. Statistical significance calculated with t-test and shown as * p < 0.05, ** p < 0.01, *** p< 0.001.*

Supplementary figure 8

A)

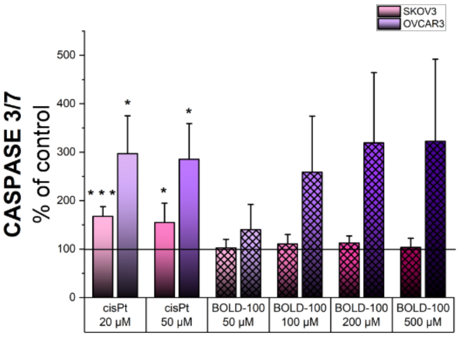

B)

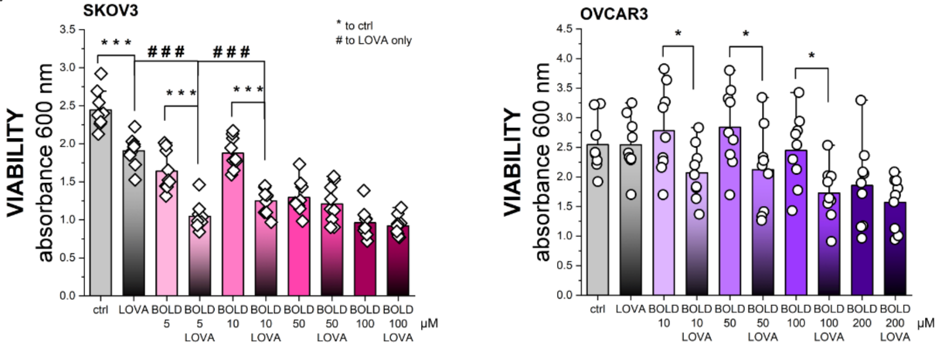

Suppl. Fig. 8 A) *Activation of Caspase3/7 in cells treated with cisPt (10 µM and 20 µM) and BOLD-100 in concentration range 50 – 200 µM for 48 h. Data expressed as percentage of solvent control.* ***B)*** *Viability of cells treated with LOVA (1 µM) and BOLD-100 (5 – 100 µM in SKOV3, 10-200 µM in OVCAR3) and their combination. Graphs show raw absorbance data. * depicts significance to solvent control, # depicts significance of combination to LOVA only. All experiments were performed in at least 3 independent biological replicates. Statistical significance calculated with t-test and shown as */# p < 0.05, **/## p < 0.01, ***/### p< 0.001.*

Supplementary figure 9

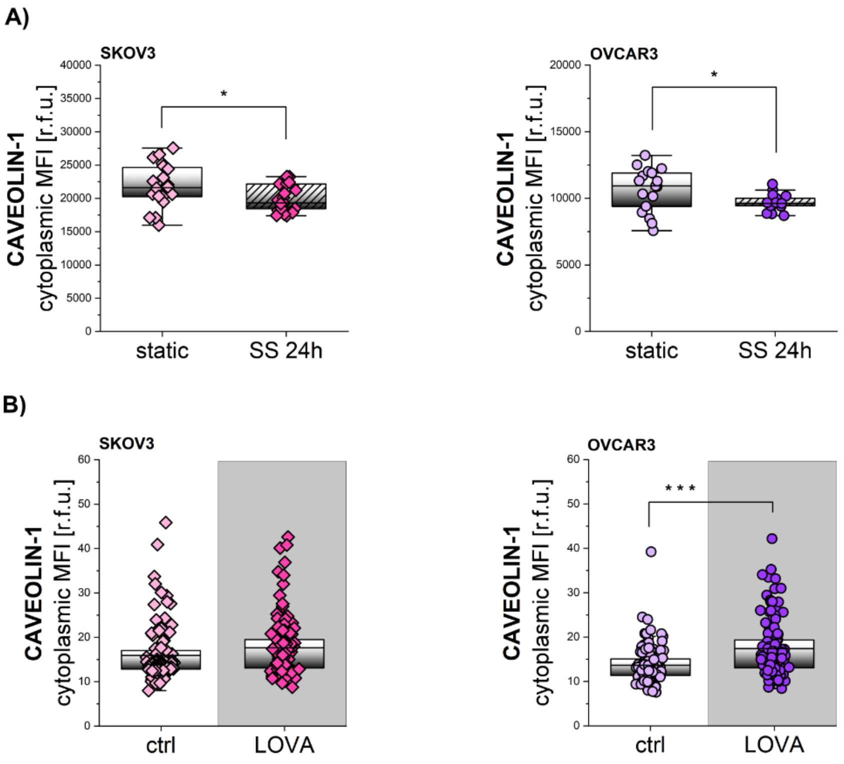

Suppl. Fig. 9 A) *Quantification of Caveolin-1 expression at the plasma membrane in cells exposed to SS for 24 h. Images were acquired with Lionheart FX automated microscope. Data represents n>15 optical fields.* ***B)*** *Quantification of Caveolin-1 expression at the plasma membrane in cells exposed to LOVA (1 µM) for 24 h. Images were acquired with LSM Zeiss 710 equipped with ELYRA PS.1 system. Data represents ROIs (1 ROI per cell). All experiments were performed in at least 3 independent biological replicates. Statistical significance calculated with t-test and shown as * p < 0.05, ** p < 0.01, *** p< 0.001.*

Supplementary figure 10

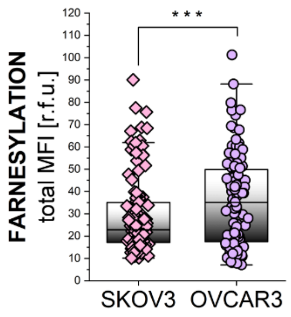

Suppl. Fig. 10 *Quantification of total level of farnesylated proteins. Primary anti-farnesylation antibody (cat. n. PA1-12554, ThermoFisher) was applied in dilution 1:1000 and secondary antibody anti-rabbit AF488 (cat. n. A21206, ThermoFisher) in dilution 1:1000. Images were acquired with LSM Zeiss 710 equipped with ELYRA PS.1 system. Data represents signal intensity of whole cell (n > 120). All experiments were performed in at least 3 independent biological replicates. Statistical significance calculated with t-test and shown as * p < 0.05, ** p < 0.01, *** p< 0.001.*
